# Health-Related Rumor Debunking on Sina Weibo in China (2009-2024): 15-Year Retrospective Infodemiology Study

**DOI:** 10.2196/91516

**Published:** 2026-09-10

**Authors:** Yuan Fang, Chengwu He, Yejinxuan Hu, Xianyun Tian

**Affiliations:** 1 College of Management Science, Chengdu University of Technology, No. 1, East Third Road, Erxianqiao, Chenghua District, Chengdu, Sichuan, 610059, China, 86 17794550089

**Keywords:** infodemiology, health misinformation, rumor debunking, social media, health communication, public health surveillance

## Abstract

**Background:**

Understanding how health-related rumor debunking evolves and spreads on social media is critical for public health communication and policy. Existing research, however, has been largely crisis-centered—dominated by studies of specific events, such as the COVID-19 pandemic—and offers limited insight into the longer-term patterns of thematic evolution, demographic targeting, and engagement dynamics of official debunking practices.

**Objective:**

This study aimed to provide an integrated understanding of official health rumor debunking in China over a 15-year period by delineating its thematic evolution, demographic disparities, and features associated with engagement and information diffusion.

**Methods:**

We collected rumor debunking posts published on Sina Weibo between 2009 and 2024. A 2-stage classification pipeline using Sentence Transformer Fine-Tuning was constructed to identify health-related debunking posts. We applied BERTopic (BERT: Bidirectional Encoder Representations from Transformers) to map the thematic landscape and used a large language model to extract demographic mentions (gender, age, and social roles), and health content referenced in the posts. Finally, we used Extreme Gradient Boosting regression models with Shapley Additive Explanations to quantify the relative contributions of predictors to engagement and information diffusion, incorporating a multidimensional feature space spanning user metadata, content characteristics, and temporal and contextual attributes.

**Results:**

Across 377,520 rumor debunking posts from 24,742 blue-verified accounts, 93,799 posts (24.8%) were health-related. Health debunking volume surged during the COVID-19 pandemic and remained elevated above prepandemic levels through 2024, although its relative share declined markedly in 2024, indicating a shift away from pandemic-centered topics, even as overall debunking activity continued to rise. Prevalent themes included vaccines, debunking reports, personal care, and sleep hygiene. Temporal trajectories fell into 3 patterns: event-driven spikes, sustained growth, and recurrent fluctuations. Demographic mentions were uneven: women were referenced more often than men, youth were the most frequently mentioned age group, and older adults were the least mentioned. Engagement was predicted primarily by source-level reach: follower count was the strongest predictor of reposts, comments, and likes alike, showing a nonlinear, threshold-like pattern in each case, whereas the secondary predictors differed across the 3 interaction types.

**Conclusions:**

This 15-year longitudinal analysis shows that health-related debunking on Sina Weibo was strongly shaped by major public health crises, surging during the COVID-19 pandemic and remaining elevated, while broadening toward routine, lifestyle-related concerns. Across all interaction types, engagement was shaped more by source reach than by the content of corrections. Although most posts addressed the general public, those with explicit demographic references revealed uneven representation across gender and age groups, with distinct health concerns linked to each. Taken together, these findings reveal a structural asymmetry in the debunking ecosystem: content is diversifying while distribution remains governed by source-level reach, suggesting that platform-level mechanisms that help credible but less-followed sources circulate corrections may be valuable for amplifying reliable health information.

## Introduction

The rapid expansion of the internet has lowered barriers to information dissemination, enabling content to circulate through online networks at unprecedented speed [1,2]. In such environments, rumors, unverified claims that spread widely in the absence of conclusive evidence, can proliferate, distort public opinion, amplify anxiety, and, in some cases, lead to serious real-world consequences [3-7].

Among the many forms of online misinformation, health-related rumors are particularly concerning because they involve issues closely tied to individual safety and public well-being [8]. At the same time, the specialized nature of medical knowledge often makes it difficult for nonexperts to assess information credibility, rendering many users vulnerable to misleading claims [9-13]. When such misleading claims take root among the public, they can disrupt public health decision-making, erode institutional trust, and produce severe real-world consequences [14,15]. During the COVID-19 pandemic, for example, false claims that consuming methanol or alcohol-based disinfectants could eliminate the virus resulted in hundreds of preventable deaths and thousands of hospitalizations worldwide [16].

To mitigate these risks, governments, public health agencies, social media platforms, and news organizations have increasingly adopted rumor debunking strategies to curb the spread of misleading health information and restore public trust [17-19]. On social media platforms, official debunking posts play an increasingly important role in countering misinformation by providing corrective information from authoritative sources and helping to mitigate the adverse effects of false claims [20,21]. Rather than merely reacting to individual falsehoods, these debunking messages function as systematic institutional interventions embedded within broader information governance structures. As such corrective communication becomes increasingly central to public health, understanding how health-related debunking operates in real-world social media environments has emerged as an urgent priority for both researchers and practitioners.

Existing research on health misinformation and rumor debunking has advanced along several complementary lines. Prior studies have examined the thematic characteristics of health misinformation, identifying recurring issue domains such as public health crises, diseases, and diet- and nutrition-related claims [22,23]. Other work has investigated who is susceptible to health misinformation, highlighting the roles of demographic and psychological factors in shaping vulnerability to misleading information [12,24]. Researchers have also explored how misinformation spreads across social media platforms, documenting diffusion dynamics and network mechanisms [5,25]. In parallel, substantial attention has been devoted to misinformation governance, including automated identification and monitoring systems [4,26], evaluations of corrective and debunking strategies [17,18], and the mechanisms and temporal durability of prebunking (inoculation) interventions [19,27]. Finally, researchers have examined audience responses to corrections, identifying factors that influence the acceptance of or resistance to debunking messages [18,28,29]. Despite this growing body of literature, several important gaps remain.

First, existing studies on health-related debunking have largely focused on specific public health crises—most notably COVID-19—thereby providing episodic snapshots rather than a longitudinal understanding of evolving discourse [15]. For example, Yang et al [30] examined rumor characteristics during the COVID-19 pandemic, whereas Sicilia et al [31] focused on the Zika virus. Although such crisis-centered approaches provide valuable insights into debunking dynamics under exceptional conditions, they are typically confined to relatively short time windows. Consequently, existing studies still lack a macro-level understanding of how health debunking themes are structured and how they evolve over time on social media platforms. Developing such a longitudinal perspective is important for informing the strategic allocation of debunking resources and supporting long-term public health governance planning [13,32,33].

Second, prior research has carefully examined who is susceptible to health misinformation. Li and Shu [34], for instance, identified demographic attributes such as gender and age as key determinants of vulnerability. Beyond demographics, cognitive traits have also been given substantial attention: Chua and Banerjee [35] highlighted the vulnerability of “naïve believers” who view knowledge as static, while Pennycook and Rand [36] suggested that susceptibility is often associated with cognitive miserliness, particularly among individuals who rely on intuition rather than analytic reasoning. However, much of this evidence is derived from self-reported surveys or small-scale experiments. As a result, existing findings may lack ecological validity and may not fully capture which demographic groups are explicitly referenced in real-world debunking narratives or what kinds of health concerns are associated with them within platform-based information ecosystems [37]. In practice, understanding which groups are explicitly mentioned as targets or affected audiences and what health risks are associated with these groups is crucial for precision debunking, improving intervention effectiveness, and optimizing resource allocation.

Third, existing research on the diffusion of health debunking information has primarily focused on differences across debunking content types, determinants of user engagement, and variations in debunking strategies. For example, Yang et al [30] examined the effects of rumor categories (dread vs wish) and rhetorical strategies, while Chua and Banerjee [35] investigated how different formats (text vs image) influence sharing intentions depending on user epistemic beliefs. Methodologically, however, this line of work predominantly relies on linear statistical models, such as regression analysis. While useful for estimating average effects, these approaches are often ill-suited for the skewed and nonlinear nature of social media diffusion data. As Sicilia et al [31] noted, online information diffusion is shaped by complex network structures and long-tail distributions of influence (eg, follower counts), conditions that frequently violate the assumptions of traditional regression models. Consequently, how multidimensional factors—spanning source, content, and contextual attributes—jointly predict different modes of public engagement remains insufficiently understood. Uncovering these nonlinear mechanisms is crucial for platforms to optimize resource allocation and enhance the visibility of corrective communication.

Against this backdrop, this study conducted a systematic analysis of health-related debunking posts published by verified accounts on Sina Weibo (Beijing Weimeng Chuangke Network Technology Co., Ltd.), covering the period from August 2009 to December 2024. Notably, although these 3 research gaps have each attracted independent scholarly attention, existing studies have rarely examined them in conjunction. Yet the 3 dimensions correspond to successive stages of the same corrective communication process, from what is produced, to whom it addresses, to how far it travels, and the overall functioning of the debunking ecosystem may depend on their interaction rather than on any single dimension alone. Through this large-scale longitudinal investigation, we therefore aimed to advance an integrated, macro-level understanding of official health debunking practices in China by addressing the following research questions (RQs):

- RQ1: What characterizes the thematic landscape of official health debunking in China, and how have these themes evolved over the past 15 years?
- RQ2: Who are the targeted and affected groups mentioned in official health rumor debunking posts, and how do the associated health risks and concerns differ across these groups?
- RQ3: What are the primary predictors of public engagement with official health debunking information, and how do their marginal contributions differ across interaction modes such as reposts, comments, and likes?

By addressing these questions, this study not only offers a comprehensive account of China’s official health debunking efforts within a rapidly evolving social media environment but also provides actionable insights for public health authorities and platform administrators seeking to strengthen precise and efficient governance of health misinformation.

## Methods

### Study Design

**Figure 1.**
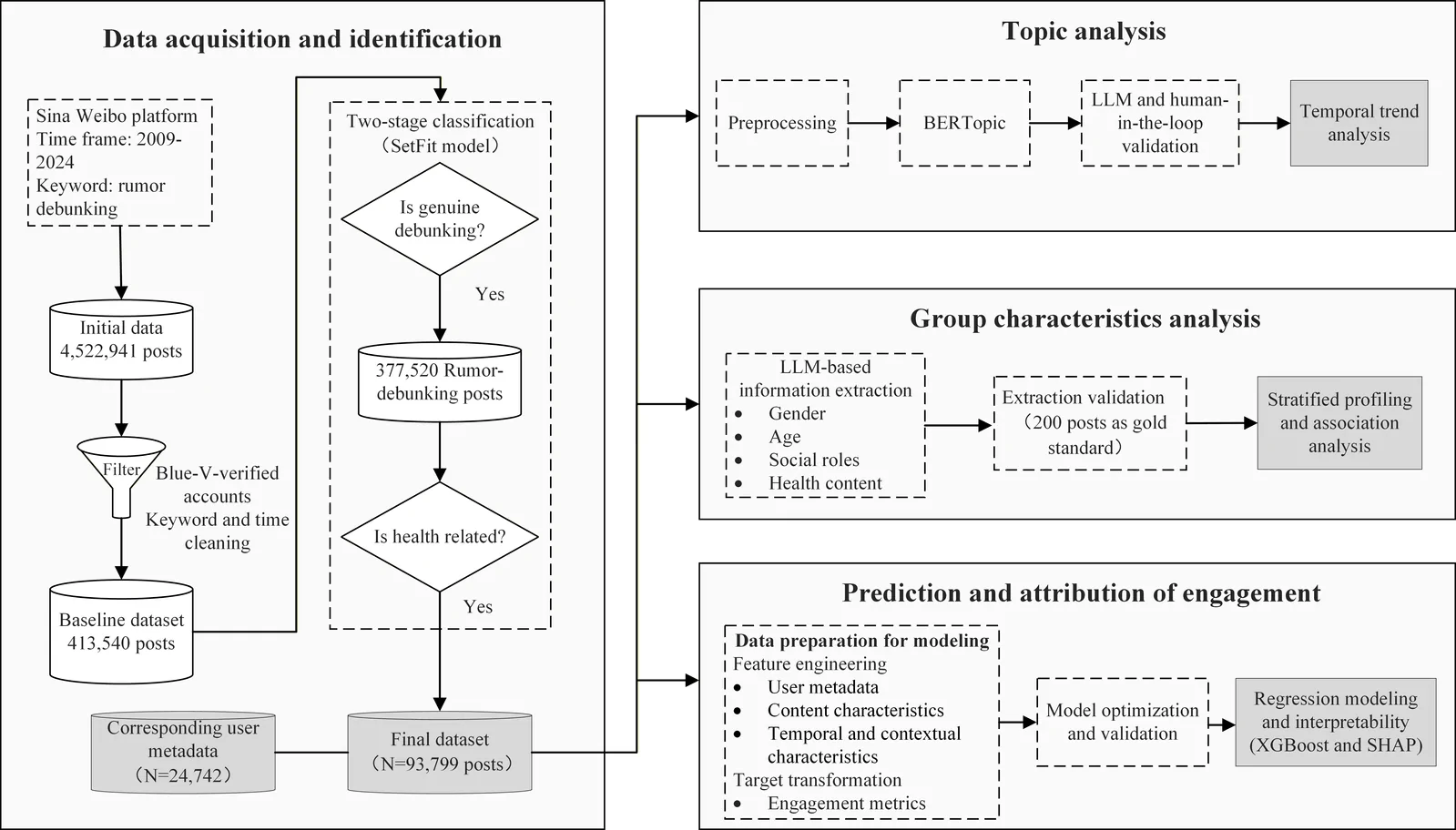
Schematic of the research framework. BERT: Bidirectional Encoder Representations from Transformers; LLM: large language modeling; SHAP: Shapley Additive Explanations; SetFit: Sentence Transformer Fine-Tuning.

This study adopted a retrospective infodemiology design to examine health-related rumor debunking posts on Sina Weibo over a period of 15 years (2009‐2024). Our analytical framework integrates natural language processing, large language models (LLMs), and interpretable machine learning to address three core dimensions: (1) the thematic evolution of health-related debunking, (2) the demographic profiles of the groups targeted or affected in debunking narratives, and (3) the key predictors associated with information diffusion and user engagement. The overall research workflow is summarized in Figure 1.

### Data Acquisition and Prefiltering

Sina Weibo is one of China’s most influential social media platforms, reporting 587 million monthly active users [38]. Its large-scale and diverse user base make it a valuable source for examining public discourse related to health rumor debunking.

Using the Chinese keyword “rumor debunking” (辟谣), we retrieved posts published between August 1, 2009, and December 31, 2024. The initial corpus comprised 4,522,941 posts authored by 24,801 users.

To improve data quality, we applied a multistep filtering pipeline. First, based on Weibo’s verification labels, we retained 493,956 posts published by “Blue V” (blue-verified) accounts, which are officially certified government agencies, media outlets, and organizational users. This restriction was motivated by prior evidence that official entities tend to provide more authoritative and effective rumor corrections [39,40]. Second, we cleaned the data by removing 80,416 posts that either did not contain the keyword or fell outside the specified time window, yielding 413,540 candidate posts. Third, we applied classifiers, as detailed in the “Classification Strategy and Model Selection” section, to identify genuine debunking posts and further categorize them into health-related versus other topics.

Subsequently, we retrieved user-level metadata using the user IDs associated with the identified genuine debunking posts. For each post, we compiled a comprehensive feature set. Post-level attributes included the text content, publication time, and engagement metrics (likes, comments, and reposts), whereas user-level metadata (when available) included user ID, nickname, profile description, verification type, follower and following counts, and membership level. Account metadata for 948 users (1723 posts) were unavailable due to account anomalies (eg, self-deactivation or platform restrictions), but the corresponding posts were retained to preserve coverage for textual and thematic analyses. Ultimately, the final dataset contained 377,520 posts from 24,742 blue-verified accounts, including 93,799 health-related rumor debunking posts and 283,721 non–health-related rumor debunking posts. For the analysis of publisher verification types, posts with missing or invalid verification-type information were excluded, resulting in a subset of 282,039 posts. Publisher verification-type distributions between health-related and non-health-related posts were compared using a Pearson chi-square test, with Cramér *V* used to quantify the effect size.

### Classification Strategy and Model Selection

#### Overview

Following standard practices in text classification [41], we adopted a 2-stage pipeline to improve task tractability and classification accuracy. Specifically, we first identified genuine rumor debunking posts from the full corpus and then classified the identified debunking posts as health related versus non–health related. This staged design reduces task complexity and enables models to learn more discriminative features, thereby improving overall performance [42,43]. To ensure the reliability of this pipeline, we relied on 2 core components: rigorous human annotation to establish high-quality gold-standard labels and systematic model evaluation to select the most robust classifier.

#### Annotation Procedure and Label Definitions

We developed the annotation guidelines through an iterative process. The first author drafted an initial guideline after reviewing 500 randomly sampled posts. Next, the first and second authors completed multiple rounds of independent annotation, each round using a newly sampled set of 500 posts generated with different random seeds. The guideline was refined after each round until disagreements were largely eliminated and the criteria stabilized.

A post was labeled as debunking if it explicitly identified specific information as a rumor. Debunking posts were further labeled as health-related if they concerned human health, including (but not limited to) psychology, diet, medical treatments, disease prevention, lifestyle habits, infectious diseases, or public health events.

Using the finalized criteria, 3 authors participated in the final annotation. An additional 500 posts were sampled using a fixed random seed, and the first 2 authors independently annotated this set to ensure reproducibility. Interannotator reliability was assessed using Krippendorff α, which accommodates different data types and numbers of coders; values above 0.80 are commonly interpreted as strong reliability [44]. After α exceeded 0.80 for both classification tasks, any remaining disagreements were resolved through discussion, with the third author mediating when necessary, to produce the final gold-standard datasets. The resulting α values were 0.835 for debunking identification and 0.909 for health topic classification.

#### Model Evaluation and Selection

To select the best-performing classifier, we evaluated a range of representative approaches in natural language processing using 5-fold cross-validation on the gold-standard datasets. We tested traditional machine learning baselines (support vector machine, random forest, Extreme Gradient Boosting [XGBoost], and logistic regression), a deep learning sequence model (attention-based bidirectional long short-term memory), and transformer-based pretrained language models (bert-base-chinese and Chinese-roberta-wwm-ext). We also evaluated Sentence Transformer Fine-Tuning (SetFit), a label-efficient framework that fine-tunes sentence transformers with relatively few labeled examples [45]. For SetFit, we compared multiple backbone models (bert-base-chinese, Chinese-bert-wwm-ext, paraphrase-multilingual-MiniLM-L12-v2, and Chinese-roberta-wwm-ext) to identify the most suitable option for our Chinese corpus, which was subsequently deployed to classify the full candidate dataset.

### Topic Analysis

#### Topic Identification

To uncover latent themes in health-related rumor debunking posts, we applied BERTopic (BERT: Bidirectional Encoder Representations from Transformers), a topic modeling framework that combines transformer-based sentence embeddings with density-based clustering [46]. Unlike probabilistic bag-of-words models such as Latent Dirichlet Allocation, which rely on document-topic distributions, BERTopic captures contextual semantic relationships at the sentence level, making it particularly suitable for short and colloquial Weibo texts [47].

Before topic modeling, we conducted light preprocessing to reduce platform-specific noise while preserving sentence structure for embedding generation. Specifically, we removed topic hashtags enclosed by “#” (eg, #health-related rumor-debunking#), user mentions starting with “@” (eg, @People’s Daily), and URLs. After cleaning, 2 posts with empty content were excluded.

BERTopic was then applied to the processed corpus. Texts were embedded using the sentence transformer model paraphrase-multilingual-mpnet-base-v2 [48]. We reduced the embedding dimensionality to 5 components using Uniform Manifold Approximation and Projection and clustered the reduced embeddings with Hierarchical Density-Based Spatial Clustering of Applications with Noise (HDBSCAN). HDBSCAN uses a soft-clustering approach that accommodates structural noise by modeling ambiguous texts as outliers (topic −1) [46]. By preventing the forced assignment of unrelated documents, this mechanism improves topic coherence and semantic interpretability.

For each cluster, we extracted the 10 most representative terms using class-based Term Frequency–Inverse Document Frequency, which estimates term importance across document clusters rather than individual documents, thereby generating robust topic-word distributions [46]. To improve interpretability, we filtered topic keywords using a publicly available Chinese stop-word list (Baidu stop-word list) and removed numerical digits (0‐9) to avoid noninformative tokens in topic descriptions.

#### Topic Validation and Labeling

To verify that the HDBSCAN outlier exclusion (topic −1) was temporally unbiased and did not distort the longitudinal dataset, we compared the month-by-month distribution of outlier versus retained posts across the 2009 to 2024 study window using Spearman rank correlation and the Kolmogorov-Smirnov test. Having confirmed the temporal robustness of the dataset, we then quantitatively evaluated the semantic quality of the retained clusters by calculating topic coherence and topic diversity scores based on the top representative keywords.

While these quantitative metrics confirmed the structural robustness of the clusters, assigning accurate labels to colloquial social media discourse requires deeper semantic comprehension. Therefore, we used a human-in-the-loop framework to finalize the topic representations. For each of the top 10 topics, we extracted the 10 highest-ranked class-based Term Frequency–Inverse Document Frequency keywords, along with the 5 most representative documents (selected based on the highest count of matched core keywords, prioritizing longer texts when match scores were equal to ensure maximum contextual richness for the LLM). We then prompted Doubao (Doubao-Seed-2.0-pro-260215), a leading Chinese LLM according to the SuperCLUE [49] benchmark, to generate preliminary academic labels using the extracted keywords and documents as context. Finally, one of the authors reviewed each generated label against the raw representative documents and the broader cluster content; any labels deemed potentially inaccurate or ambiguous were discussed and refined through team consensus among the co-authors to ensure semantic accuracy.

#### Temporal Trend Analysis

To examine how health-related rumor debunking themes evolved over the study period, we assigned each post its corresponding BERTopic topic label and aligned these labels with publication time stamps. We then computed and visualized the topic frequency over time, which enabled us to identify shifts in prominence and the emergence of new themes across years.

### Group Characteristics Analysis

#### Automated Information Extraction via LLMs

Given the finite resources available for large-scale information governance, accurately identifying vulnerable groups is critical for efficiently targeting health-related rumor debunking. To characterize the populations referenced in debunking posts and examine their associations with specific health topics, we used DeepSeek-V3.2, an LLM with strong Chinese language understanding and instruction-following capability [50], to build a standardized information extraction pipeline. The model was accessed via an OpenAI-compatible API and applied to the full dataset in batch processing mode. We designed an extraction schema comprising 4 key variables to capture the multidimensional nature of digital health vulnerability: gender and age provide the core demographic strata; social roles (eg, occupation) capture contextual vulnerability beyond biological attributes; and health content reflects the specific medical concerns linked to misinformation.

To transform diverse textual features into quantifiable demographic variables, we enforced strict standardization through a rule-embedded system prompt. We designed a comprehensive instruction set that directs the model to map extracted features based on predefined criteria. For gender, diverse referents ranging from specific terms such as “Mr.” to implicit relational terms such as “mother” are mapped to unified “male” or “female” categories. Regarding age, we established a hybrid classification framework anchoring definitions in legal and medical standards. Specifically, minors (aged <18 y) and the older adults (aged ≥60 y) follow the strict definitions of the Law on the Protection of Minors [51] and the Law on Protection of the Rights and Interests of the Elderly of the People’s Republic of China [52], respectively. The intermediate spectrum is further stratified into youth (aged 18-44 y) and middle-aged (aged 45-59 y), drawing upon epidemiological evidence regarding the onset of chronic noncommunicable diseases [53].

Furthermore, we addressed the structural complexity of social media content, particularly “listicle” style posts where a single text often debunks multiple unrelated rumors simultaneously. To resolve this, we integrated structural demonstrations (few-shot examples) into the prompt. This guided the model to parse complex texts and segment distinct semantic units, ensuring that social roles and specific health content were extracted as structured lists corresponding to each subtopic, thereby preserving semantic granularity and completeness.

#### Validation of the Extraction Framework

Before large-scale deployment, we validated the reliability of this extraction framework using a 200-post gold-standard set annotated independently by 2 coders. Interannotator agreement (IAA) was first calculated to establish the reliability of the human annotations. The model’s extraction performance was assessed by comparing the outputs of DeepSeek-V3.2 with the human-adjudicated ground truth. Evaluation metrics were selected to match the structural characteristics of each extracted attribute. For standardized categorical variables, specifically the mapped gender and age groups, Krippendorff α was used to measure IAA, and the Macro *F*_1_-score was used to evaluate model performance. For unordered entity lists, such as raw demographic mentions and identity labels, a set-based *F*_1_-score was applied. Finally, for free-text health narratives, BERTScore was used to assess semantic similarity beyond surface-level lexical overlap. The validation results demonstrated high IAA across all dimensions, and the pipeline achieved strong alignment with the adjudicated benchmark, thereby supporting its robustness for extracting group mentions and health content from the full corpus (n=93,799). Detailed validation metrics, including overall performance evaluations and category-specific metrics for gender and age variables, are provided in Tables S1-S3 in Multimedia Appendix 1.

#### Demographic and Thematic Association Analysis

On the basis of the standardized outputs returned by the validated pipeline, we conducted descriptive and association analyses to characterize group mentions and their topical distributions. We first calculated the frequency of extracted gender and age groups across the top BERTopic topics. We then performed word frequency analysis on the extracted social roles and health content to summarize salient health narratives for each demographic subgroup using Chinese word segmentation and stop-word filtering. The most frequent keywords were computed to identify prominent social identities and compare differences in health concerns across demographic strata.

### Prediction and Explanation of Information Diffusion via XGBoost and Shapley Additive Explanations

#### Overview

To examine the complex factors associated with the propagation of health-related rumor debunking posts, it was necessary to account for the nonlinear dynamics and diminishing returns inherent in social media diffusion [54]. To capture these patterns while maintaining interpretability, we adopted an interpretable machine learning framework combining XGBoost Regressor for predicting continuous engagement outcomes [55], with SHAP (Shapley Additive Explanations) [56] for model interpretation. This framework enabled us to estimate the expected magnitude of user engagement while quantifying the marginal contributions of source characteristics, content attributes, and contextual cues, thereby facilitating a more transparent understanding of the mechanisms underlying rumor debunking diffusion [57].

#### Feature Engineering and Target Variable Transformation

To mitigate potential omitted-variable bias and capture the multidimensional context of information diffusion on social media platforms, we engineered features across 3 major dimensions: user metadata, content characteristics, and temporal and contextual attributes.

First, regarding user metadata, we included account verification type and membership category as categorical features, while ordinal membership ranks were label encoded. To reduce skewness and stabilize variance, highly skewed account-level metrics—including follower count, following count, and status count—were transformed using a base-10 logarithm.

Second, for content characteristics, we incorporated 4 primary dimensions: thematic categories, multimedia elements, text length, and sentiment polarity. Thematic categories were derived from BERTopic, and multimedia elements were represented by the presence of attached images and videos. Regarding text length, it was captured through 2 distinct features: a log-transformed continuous variable to measure overall length and a binary indicator for posts exceeding 140 characters. This design accounts for Weibo’s historical character limits and the platform’s current interface mechanism, in which longer posts are truncated and require users to click “expand” to view the full content—an additional interaction step that may introduce friction and influence user engagement behaviors.

To capture emotional attributes, sentiment polarity for the full corpus (n=93,799) was first classified into 3 categories (positive, neutral, and negative) using the Gemma4:e4b LLM (Google DeepMind) through prompt-based annotation. To evaluate the reliability of this automated annotation, we then constructed a silver-standard reference dataset—a high-quality reference set generated primarily through automated multimodel consensus, with human adjudication reserved for cases of full disagreement—through stratified sampling: 500 posts were randomly drawn from each of the 3 Gemma4:e4b-classified categories (1500 in total) to ensure adequate evaluation coverage across all sentiment classes, including the typically less frequent ones. These 1500 posts were independently reannotated using identical prompts by 3 Chinese LLMs—Doubao (Doubao-Seed-2.0-pro-260215 and ByteDance), Kimi (Kimi-K2.5-Thinking and Moonshot AI), and Qwen (Qwen3.5-397B-A17B-Thinking and Alibaba Cloud)—which had no access to the Gemma4:e4b labels. Intermodel consistency among the 3 evaluator models was assessed using the ordinal Krippendorff α coefficient, and final silver-standard labels were determined through majority voting, with human adjudication applied in cases of complete disagreement among the 3 models. Agreement between the Gemma4:e4b annotations and the silver-standard labels was subsequently evaluated using the Quadratically Weighted Cohen κ (QWK) coefficient, which accounts for the ordinal structure of sentiment polarity.

Finally, to capture temporal and contextual characteristics of posting behavior, we engineered categorical features representing posting hour, season, weekend status, and posting device source, which on Weibo is publicly visible and reflects the operational context of the posting behavior (eg, mobile devices vs professional web management clients). To reduce feature sparsity and avoid overfitting to infrequent platforms, only the 10 most common device sources were retained as independent categories, while all remaining long-tail sources were grouped into an “Other” category.

Regarding the target variables (reposts, comments, and likes), social media engagement metrics typically exhibit highly skewed long-tail distributions. To stabilize variance and reduce the disproportionate influence of extreme outliers, all engagement counts were transformed using a base-10 logarithmic transformation (log₁₀(x+1)). These transformed engagement metrics served as the continuous target variables for the regression models.

#### Model Optimization and Validation

We selected XGBoost as the primary modeling approach because gradient-boosted decision trees are well suited to capturing complex interactions and diminishing returns common in diffusion processes [58]. In addition, prior benchmarks on tabular prediction tasks have shown that tree-based ensembles often provide strong accuracy and computational efficiency at this scale [59]. As reposts, comments, and likes represent different modes of user engagement, model optimization was conducted independently for each target variable. Using the Optuna framework, we performed independent hyperparameter optimization (eg, tuning learning rate, max depth, and subsample) to minimize the validation root-mean-squared error (RMSE) for each target variable, yielding 3 distinct models. To prevent data leakage from users with multiple posts, these models were evaluated using a rigorous 5-Fold GroupKFold Cross-Validation strategy, grouped by user ID. Model performance was quantified using *R*² and RMSE.

#### Interpreting Feature Contributions Using SHAP

To interpret the fitted regression models, we used SHAP, a game-theoretic framework that decomposes continuous predictions into feature-level contributions [56]. SHAP supports 2 complementary forms of explanation in our study: (1) global feature importance, which identifies the most influential predictors across the corpus; and (2) dependence plots, which visualize nonlinear effects and reveal potential thresholds where a predictor’s marginal contribution changes sharply [57]. Together, these analyses provide an interpretable account of what is associated with engagement magnitude and diffusion in health-related rumor debunking posts.

### Ethical Considerations

All data were collected exclusively from publicly accessible posts on the Sina Weibo platform. The study involved retrospective analysis of publicly available content and did not include any direct interaction with or intervention involving human participants. To protect user privacy, analyses were conducted at an aggregate level, and any personally identifiable information was removed or anonymized prior to analysis.

## Results

### Two-Stage Classification Results

We constructed the final analytical sample using a 2-stage classification pipeline. In stage 1 (debunking identification), SetFit with the Chinese-roberta-wwm-ext backbone achieved the best performance among 11 candidate models, with a weighted *F*_1_-score of 0.8973, indicating strong discriminative performance (Table 1). Applying this model to the 413,540 candidate posts identified 377,520 rumor debunking posts.

**Table 1.** Performance comparison of classification models for debunking identification (stage 1).

Model	Accuracy	Precision	Recall	*F*_1_-score (macro)	*F*_1_-score (weighted)
Traditional machine learning					
SVM^a^	0.7000	0.8971	0.7262	0.5888	0.7342
Random forest	0.6100	0.8571	0.6429	0.4994	0.6594
XGBoost^b^	0.6100	0.8814	0.6190	0.5215	0.6614
Logistic regression	0.8100	0.8421	0.9524	0.4945	0.7661
Standard deep learning					
Att-BLSTM^c^	0.6100	0.9245	0.5833	0.5481	0.6618
bert^d^-base-chinese	0.8800	0.9186	0.9405	0.7647	0.8767
Chinese-roberta^e^-wwm-ext	0.9000	0.9302	0.9524	0.8039	0.8973
Few-shot learning					
SetFit^f^ (Chinese-bert-wwm-ext)	0.8900	0.9195	0.9524	0.7782	0.8853
SetFit (bert-base-chinese)	0.8900	0.9101	0.9643	0.7645	0.8814
SetFit (paraphrase-multilingual-MiniLM-L12-v2)	0.8900	0.9195	0.9524	0.7782	0.8853
SetFit(Chinese-roberta-wwm-ext)^g^	0.9000	0.9302	0.9524	0.8039	0.8973

a SVM: support vector machine.

b XGBoost: Extreme Gradient Boosting.

c Att-BLSTM: Attention-based Bidirectional Long Short-Term Memory.

d BERT: Bidirectional Encoder Representations from Transformers.

e RoBERTa: a robustly optimized BERT pretraining approach.

f SetFit: Sentence Transformer Fine-Tuning.

g Best performing model overall.

In stage 2 (health topic classification), the same SetFit (Chinese-roberta-wwm-ext) configuration again performed best, reaching a weighted *F*_1_-score of 0.9405, reflecting robust classification performance (Table 2). When applied to the 377,520 debunking posts, it yielded 93,799 health-related posts (24.8%) and 283,721 non–health-related posts (75.2%).

**Table 2.** Performance comparison of classification models for health topic classification (stage 2).

Model	Accuracy	Precision	Recall	*F*_1_-score (macro)	*F*_1_-score (weighted)
Traditional machine learning					
SVM^a^	0.7500	0.6190	0.4333	0.6710	0.7355
Random forest	0.6600	0.4412	0.5000	0.6094	0.6656
XGBoost^b^	0.6100	0.3953	0.5667	0.5793	0.6248
Logistic regression	0.7600	0.6500	0.4333	0.6800	0.7440
Standard deep learning					
Att-BLSTM^c^	0.7000	0.5000	0.2667	0.5765	0.6680
bert^d^-base-chinese	0.9200	0.8667	0.8667	0.9048	0.9200
Chinese-roberta^e^-wwm-ext	0.9100	0.8387	0.8667	0.8939	0.9104
Few-shot learning					
SetFit^f^ (Chinese-bert-wwm-ext)	0.9200	0.8929	0.8333	0.9029	0.9192
SetFit (bert-base-chinese)	0.9100	0.8889	0.8000	0.8896	0.9086
SetFit (paraphrase-multilingual-MiniLM-L12-v2)	0.9400	0.9615	0.8333	0.9256	0.9387
SetFit (Chinese-roberta-wwm-ext)^g^	0.9400	0.8750	0.9333	0.9299	0.9405

a SVM: support vector machine.

b XGBoost: Extreme Gradient Boosting.

c Att-BLSTM: Attention-based Bidirectional Long Short-Term Memory.

d BERT: Bidirectional Encoder Representations from Transformers.

e RoBERTa: a robustly optimized BERT pretraining approach.

f SetFit: Sentence Transformer Fine-Tuning.

g Best performing model overall.

### Temporal and Source Characteristics of Health-Related Rumor Debunking on Weibo

Using the health-related debunking posts identified by the 2-stage pipeline, we examined temporal trends and source composition from 2009 to 2024.

#### Temporal Patterns of Health-Related Rumor Debunking

**Figure 2.**
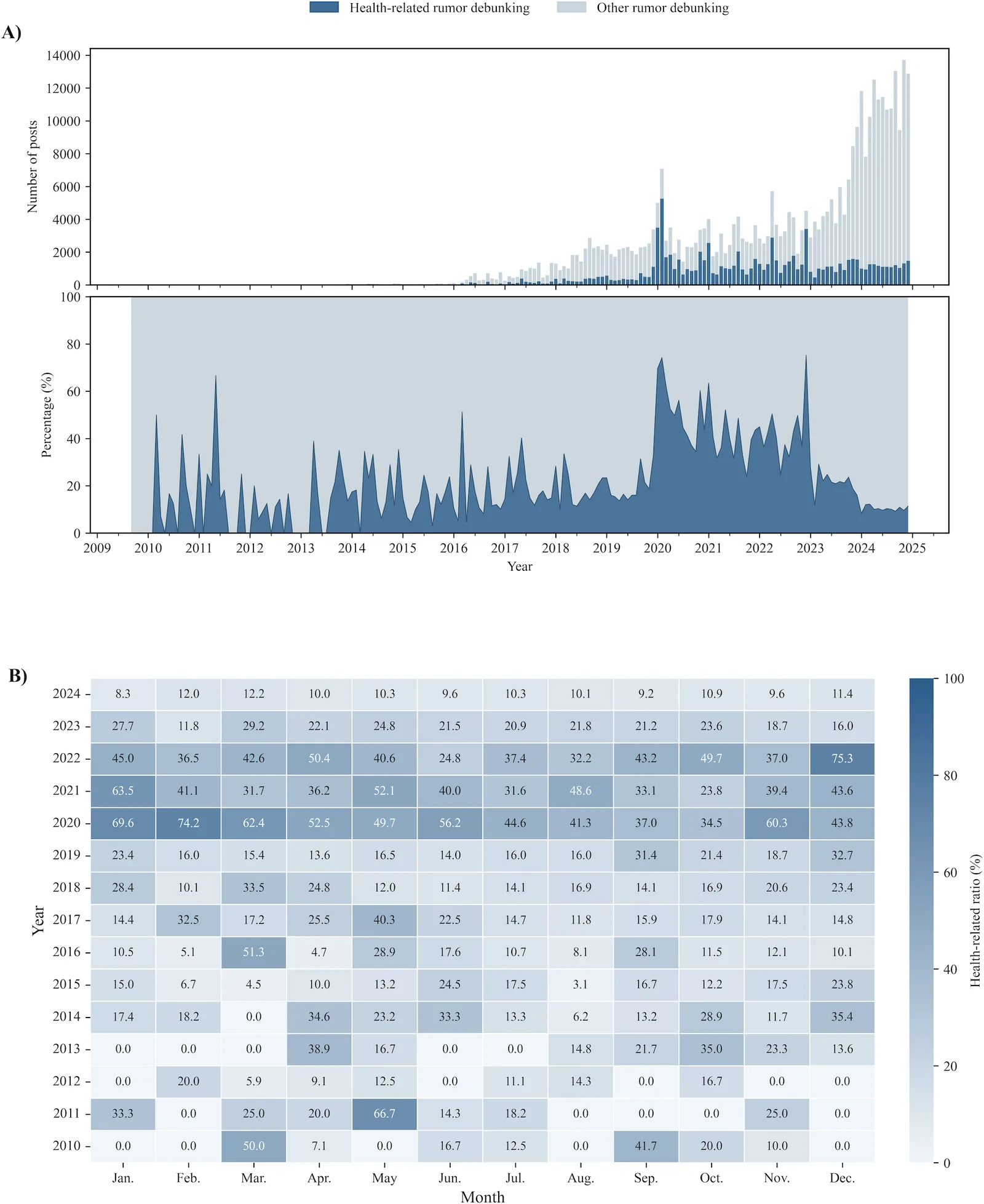
Temporal patterns of rumor debunking posts on Sina Weibo. (A) Annual post volume and the percentage of health-related content (2009‐2024). (B) Heatmap of the monthly intensity of health-related content (2010‐2024). Data from 2009 were excluded from the heatmap due to incomplete temporal coverage (available only from August to December).

From 2009 to 2024, the overall volume of rumor debunking posts increased substantially, with 2 pronounced peaks in 2020 and 2024 (Figure 2A). Importantly, these peaks differed in topical composition. The 2020 peak was characterized by an unprecedented concentration of health-related debunking posts, temporally aligned with the outbreak of the COVID-19 pandemic. In contrast, the 2024 peak was largely composed of non–health-related topics, indicating that the overall expansion of debunking activity did not translate into a parallel increase in health-related content.

To further clarify how health-related debunking responded to major public health events, we examined monthly variation in the share of health-related posts (Figure 2B). The proportion rose sharply to 69.6% in January 2020 and 74.2% in February 2020, corresponding to the initial outbreak period. A second major spike occurred in December 2022 (75.3%), coinciding with the nationwide relaxation of COVID-19 containment policies and heightened public concern about infections and medical supplies. Although the largest spikes clustered in winter months, elevated proportions were also observed in other periods (eg, April 2022: 50.4%; May 2021: 52.1%; and May 2011: 66.7%), indicating that intensified health-related debunking was not confined to winter months and is unlikely to be explained by fixed seasonality alone.

Beyond these event-driven surges, we observed a structural shift in baseline activity following the COVID-19 outbreak. As shown in Figure 2A, health-related debunking remained consistently higher during 2020 to 2024 than in the prepandemic period (2009 to 2019), indicating sustained activity in this domain throughout the postpandemic period.

Despite this sustained volume, the relative predominance of health topics did not persist into 2024. Although total rumor debunking activity reached its highest level in that year, the health-related share declined markedly and remained between 8.3% and 12.2% throughout the year. While the absolute volume remained significant, in relative terms, the proportion approached the lower levels observed during the prepandemic period (2014‐2019). Taken together, these results suggest that while rumor refutation remained highly active overall, the platform’s debunking emphasis shifted away from pandemic-centered health topics in 2024.

#### Sources of Rumor Debunking Posts

To characterize the actors involved in health-related rumor debunking, we first examined the verification types of publishers within the health domain (Table 3). Government-verified accounts accounted for the largest share of health-related debunking, contributing 43,207 (64.69%) posts. Media accounts ranked second (n=10,126, 15.16%), followed by institutions (n=5632, 8.43%) and enterprises (n=5380, 8.05%). Campus and nonprofit organizations accounted for only a marginal share, together contributing less than 4% of health-related posts.

**Table 3.** Comparison of verified user types between health-related and non–health-related rumor debunking posts^a^. Posts without valid verification-type labels (n=95,481) were excluded from this analysis.

Verified user type	Health-related posts (n=66,796), n (%)	Non–health-related posts (n=215,243), n (%)	Total (N=282,039), n (%)
Government	43,207 (64.69)	127,824 (59.39)	171,031 (60.64)
Media	10,126 (15.16)	51,391 (23.88)	61,517 (21.81)
Institution	5632 (8.43)	20,039 (9.31)	25,671 (9.10)
Enterprise	5380 (8.05)	14,841 (6.89)	20,221 (7.17)
Campus	2438 (3.65)	1119 (0.52)	3557 (1.26)
Nonprofit	13 (0.02)	29 (0.01)	42 (0.01)

a The distribution of user types differs significantly between health-related and non–health-related topics (*χ*2_1_=599.5, N=282,039; *P*<.001; Cramér V=0.05).

To contextualize this source structure, we compared it with the distribution observed in non–health-related rumor debunking. Government accounts also constituted the largest contributor outside the health domain (59.39%). Although the overall source hierarchy was similar across domains, government output was more concentrated in health-related debunking: the government-to-media ratio was approximately 4.3 in health-related posts versus 2.5 in non–health-related posts.

We further examined whether the share of government-verified publishers differed between health-related and non–health-related rumor debunking posts by conducting a Pearson chi-square test on publisher type (government vs nongovernment) across the 2 domains. The difference was statistically significant (*χ*²_1_=599.5, n=282,039; *P*<.001); however, the effect size was small (Cramér *V*=0.05), suggesting that government accounts were only modestly more prevalent in health-related debunking than in non–health-related debunking.

### Topic Modeling and Temporal Patterns

#### Topic Modeling Results

Topic modeling was conducted on 93,797 health-related rumor debunking posts after text preprocessing. BERTopic identified 352 topics. Sensitivity analysis confirmed that the exclusion of outlier documents did not systematically distort temporal patterns: the monthly post counts of outliers and retained posts were highly synchronized (Spearman ρ=0.95; *P*<.001), and their monthly distributions were statistically indistinguishable (Kolmogorov-Smirnov *D*=0.04; *P*=.99). Quantitative evaluation further demonstrated high clustering quality on the retained documents, yielding a topic coherence score of 0.6589 and a topic diversity score of 0.8043. As established measures of semantic consistency [60] and topic distinctiveness [61], these scores exceed the common empirical reference values of 0.55 and 0.70, respectively, indicating strong semantic consistency and high topic distinctiveness. Building upon these mathematically robust clusters, our human-in-the-loop framework generated the final interpretable labels. Table 4 reports the 10 most prevalent topics, including post counts, corpus proportions, and representative keywords (translated).

**Table 4.** The 10 most prevalent topics identified from health-related rumor debunking posts on Sina Weibo^a^.

Rank	Topic	Posts, n (%)	Top keywords (translated)
1	Vaccine	1140 (1.22)	Vaccination, vaccine, first, dose, Guangzhou, injection, second, later, no longer, month
2	Debunking report	1031 (1.10)	Zhao (surname), police, according to law, impersonation, administrative detention, epidemic-related, investigation, spread, egregious, reselling
3	Personal care	867 (0.92)	Skin, hair loss, cosmetic facial mask, hair follicles, hair, sunscreen, skin care, silicone oil, shampoo, hair washing
4	Sleep hygiene	733 (0.78)	Sleep, staying up late, nap, falling asleep, exercise, before bed, sleeping well, regular, sleep enough, quality
5	Food myths	721 (0.77)	Food incompatibility, food, 1935, widespread, everything is normal, discard, alarmist, peanut, animal, chestnut
6	PPE^b^ myths	704 (0.75)	Protective mask, pulmonary nodules, ethylene oxide, causing, Ye Xianwei, doubts, Guizhou Province, clarification, long-term, medical department
7	Local misinformation	659 (0.70)	Longnan, Gansu, municipal party committee, cyberspace administration, all-out, online rumors, makeshift hospital, Wuhan, Chengdu Middle School, Shijiazhuang
8	Communicable diseases	656 (0.70)	Novel, coronavirus, all, pneumonia, layered, prevent, antiviral, vinegar fumigation, prevention, salt water
9	Nutrition and lifestyle myths	653 (0.70)	Weight loss, skipping meals, dinner, eating dinner, obesity, carbohydrates, diet, autophagy, staple food, daily
10	Vision and eye health myths	619 (0.66)	Myopia, eyes, National Eye Care Day, prescription, vision, surgery, wearing glasses, presbyopia, glasses, myopia

a Topics are ranked in descending order of post count. Representative keywords were translated from Chinese. Under the current BERTopic settings, 46,616 (49.70%) posts were classified as topic −1 by Hierarchical Density-Based Spatial Clustering of Applications with Noise (outliers) and were excluded from topic-specific clusters.

b PPE: personal protective equipment.

Overall, topic prevalence was highly dispersed rather than dominated by a small set of themes. Even the most frequent topic (“vaccine”) accounted for only 1.22% of the corpus, and the remaining top-ranked topics each contributed similarly small shares. To facilitate interpretation, we grouped the top 10 topics into 2 broader domains: public health and crisis-related governance, as well as lifestyle and routine health management.

The first domain centers on infectious diseases, preventive measures, and crisis communication. For example, “vaccine” (rank 1) includes terms related to vaccination schedules and dosage, while “communicable diseases” (rank 8) and “personal protective equipment (PPE) myths” (rank 6) capture discussions involving pathogens and protective practices. In addition, some clusters, such as “debunking report” (rank 2) and “local misinformation” (rank 7), feature governance- and enforcement-related keywords (eg, “police,” “investigation,” and place names), suggesting that part of health-related rumor debunking is embedded within broader information governance and administrative enforcement contexts.

The second domain reflects recurring concerns in everyday health management. “Personal care” (rank 3) and “sleep hygiene” (rank 4) relate to appearance- and lifestyle-oriented issues, while diet-related misinformation appears in both “food myths” (rank 5) and “nutrition and lifestyle myths” (rank 9). “Vision and eye health myths” (rank 10) focuses on myopia correction and eye protection. Taken together, these results indicate that health-related rumor debunking on Weibo spans both crisis-oriented public health topics and a wide range of routine concerns about personal well-being.

#### Temporal Patterns of Major Topics

**Figure 3.**
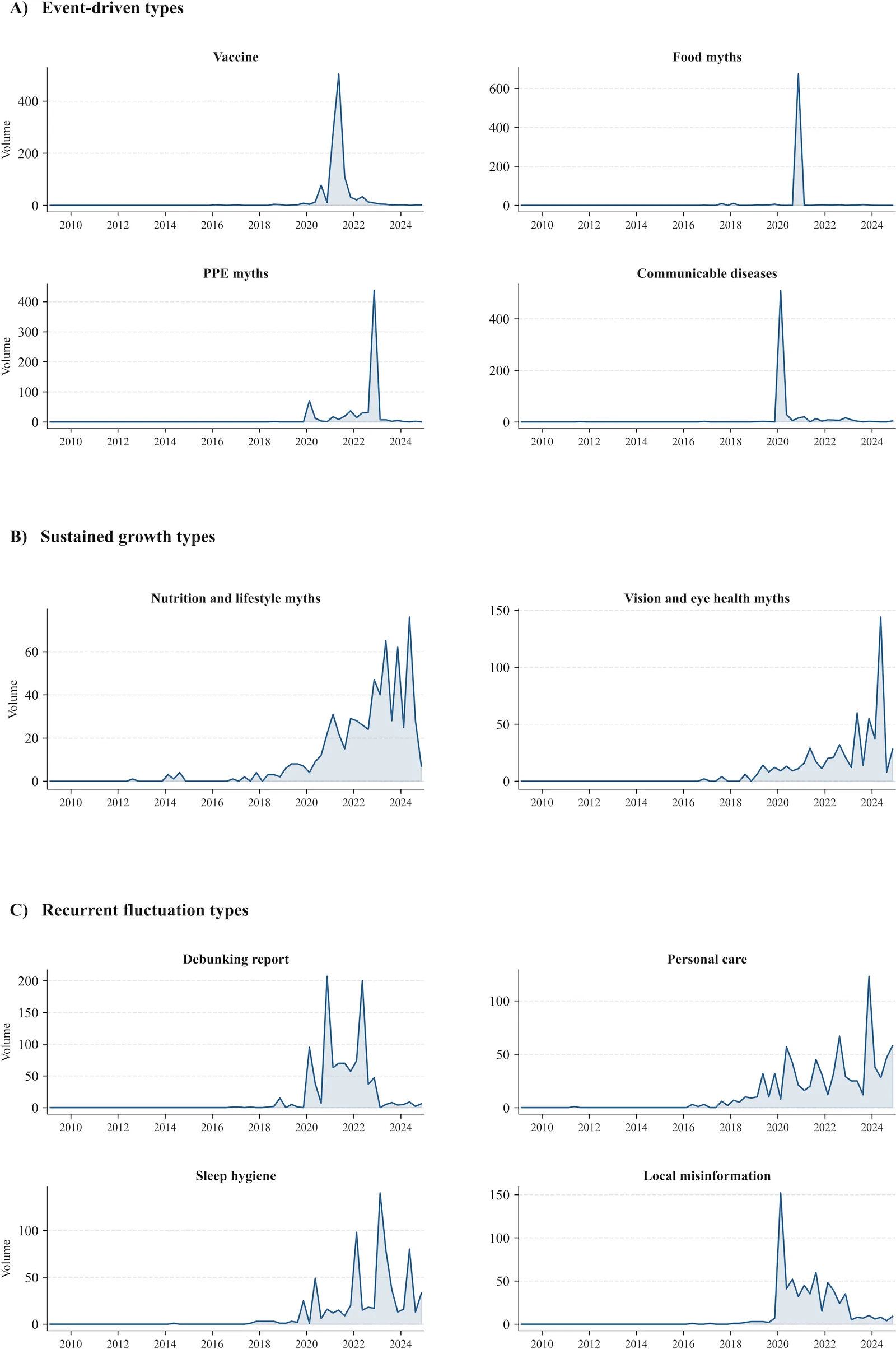
Temporal patterns of the top 10 health-related rumor debunking topics on Weibo. The time axis begins in 2010 because the earliest post among the top 10 topics was published on March 8, 2010. PPE: personal protective equipment.

Figure 3 presents the temporal trajectories of post volume across the top 10 topics. Topic activity remained relatively low before 2019 but increased markedly thereafter. Overall, the temporal dynamics of these topics fall into 3 archetypes that reflect different attention-mobilization patterns in health-related debunking: event-driven spikes, sustained growth, and recurrent fluctuations.

The first archetype, event-driven spikes (eg, vaccine, food myths, PPE myths, and communicable diseases), is defined by long periods of low activity punctuated by short-lived, high-intensity surges. These topics respond sharply to external shocks and then quickly regress toward baseline levels. For instance, vaccine-related debunking remained marginal before 2020 but rose steeply during the pandemic period, peaking around mid-2021 before declining again. Similarly, PPE myths and communicable diseases exhibit synchronized spikes in early 2020, consistent with the early phase of COVID-19–related uncertainty. Food myths differ slightly in that it shows a concentrated, single-pulse peak in 2021 rather than repeated waves.

In contrast, the second archetype, sustained growth (eg, nutrition and lifestyle myths and vision and eye health myths), shows a gradual upward trajectory with mild fluctuations, rather than abrupt shock-driven surges. These topics increase gradually over time, suggesting an expanding and persistent demand for correction in routine health management. Vision and eye health myths is particularly illustrative, rising from near-zero levels around 2016 and continuing upward to its highest activity in 2024, indicating steadily growing attention in debunking practice.

The third archetype, recurrent fluctuations (eg, debunking report, personal care, sleep hygiene, and local misinformation), differs from the other 2 patterns in that attention is repeatedly reactivated rather than driven by a single shock or a gradual rise. These topics show irregular volatility with multiple recurring peaks, reflecting episodic bursts of debunking activity over time. Although their overall activity increased after 2019, the repeated rises and declines suggest that attention cycles are triggered intermittently rather than accumulating steadily.

### Analysis of Extracted Information

#### Overall Frequencies of Mentioned Groups

Table 5 presents the distribution of target audiences identified through post-level targeting feature analysis. The majority of posts were classified as either nonoriented or universally oriented, accounting for 91.31% (n=85,648) of the 93,799 posts for gender and 87.64% (n=82,205) for age. This indicates that most health rumor debunking content was not explicitly tailored to specific demographic groups.

**Table 5.** Distribution of specific target audiences in health rumors^a^.

Demographic characteristics	Count, n (%)
Gender	
Female	5352 (5.71)
Male	2799 (2.98)
Age group	
Minor (<18 y)	4878 (5.20)
Youth (18‐44 y)	5020 (5.35)
Middle aged adults (45‐59 y)	4058 (4.33)
Older adults (≥60 y)	3590 (3.83)

a Age groups were categorized based on legal definitions and epidemiological evidence. Statistics are based on unique posts. For each demographic category, the count represents the number of unique posts containing a specific targeting feature. A single post may mention or target multiple age groups and therefore may be counted in more than one specific age-group category. Posts with no specific demographic features (“nonoriented”) or with all demographic groups mentioned (“universally oriented”) were grouped together and excluded from the specific demographic categories.

Among posts exhibiting explicit demographic orientation, clear disparities were observed. Female-oriented posts (n=5352, 5.71%) substantially outnumbered male-oriented posts (n=2799, 2.98%). With respect to age, youth constituted the most frequently targeted group (n=5020, 5.35%), followed by minors (n=4878, 5.20%) and middle-aged adults (n=4058, 4.33%). In contrast, older adult-oriented posts were the least prevalent among the defined age categories (n=3590, 3.83%).

#### Associations Between Health Content and Specific Groups

**Figure 4.**
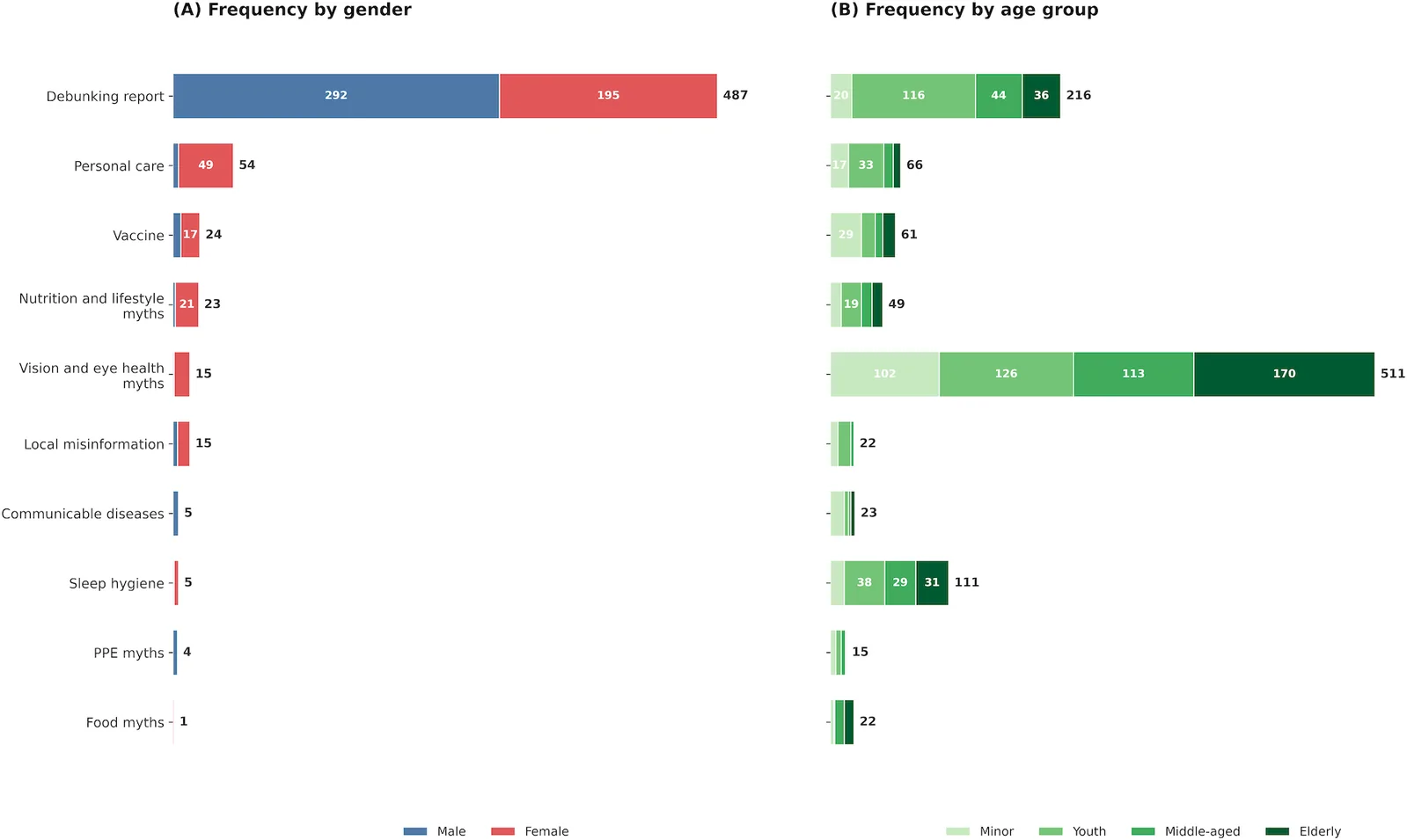
Frequency of group mentions in different topics. The total frequency of mentions per topic is shown to the right of each bar, while segment labels indicate specific subgroup counts. Labels are omitted for smaller segments for visual clarity. Detailed statistics are provided in Multimedia Appendix 2. PPE: personal protective equipment.

Figure 4 illustrates the distribution of gender- and age-referenced mentions across the top 10 BERTopic-derived themes in health-related debunking posts. Overall, both demographic dimensions exhibited marked topic-level variation, indicating that references to specific populations were unevenly distributed across thematic contexts.

In terms of gender, the aggregate frequency of mentions across the top 10 topics was relatively balanced between men (n=321) and women (n=312). However, distinct disparities emerged at the thematic level. The topic “debunking report” accounted for the largest share of gendered references for both genders, with a higher frequency in posts mentioning men (n=292) than women (n=195). In contrast, lifestyle-related topics showed a pronounced skew toward female mentions. “Personal care” was overwhelmingly associated with women (n=49) compared with men (n=5), and “nutrition and lifestyle myths” similarly exhibited a female-dominant pattern (women: n=21 and men: n=2). Conversely, men appeared slightly more frequently in technical or acute health topics such as “communicable diseases” and “PPE myths,” although the absolute numbers for these categories were low.

Age-related references displayed a more differentiated thematic distribution. Overall, youth (n=367) and older adults (n=280) were the most frequently mentioned age groups. “Vision and eye health myths” emerged as the most significant topic in the context of age-specific mentions, accumulating the highest aggregate frequency (n=511). This topic showed broad relevance across the lifespan, ranging from the older adults (n=170) and youth (n=126) to the middle-aged adults (n=113) and minors (n=102), indicating that eye health is a concern shared across age groups rather than confined to any single generation. The “youth” group dominated the “debunking report” category (n=116), significantly outpacing other age groups. In addition, “personal care” was primarily associated with youth (n=33) and minors (n=17), with comparatively few references to the older adults (n=7). The older adult group showed notable representation in “sleep hygiene” (n=31), ranking second only to youth (n=38).

Comparing gender- and age-based references reveals differences in how demographic attributes were distributed across topics. “Vision and eye health myths” accounted for a substantial proportion of age-related mentions but contained relatively few gender-specific references (total gender mentions=15). In contrast, “debunking report” posts exhibited a high density of gender references (n=487) while containing comparatively fewer age-specific mentions than the vision-related topic. Together, these patterns indicate that age and gender were emphasized in different thematic contexts within health-related debunking posts.

#### The Association Between Groups and Specific Topics

To characterize health narratives associated with different populations, we analyzed the most salient keywords linked to each demographic group (Multimedia Appendix 3). Across all strata, pandemic-related terms—most notably “pandemic,” “pneumonia,” and “infection”—were consistently prominent, establishing a shared thematic baseline for health rumor debunking during the study period. Beyond this common context, distinct gender- and age-specific keyword patterns were observed.

The female-associated keyword profile was dominated by terms related to reproductive and maternal health, including “uterus,” “ovary,” “gestational period,” “fetus,” and “pregnancy brain.” In contrast, the male-associated profile exhibited a markedly different focus, featuring keywords related to acute cardiovascular events and pandemic control measures, such as “cardiac arrest,” “heart,” “choking,” “lockdown,” and “quarantine.” Reproductive health–related terms were largely absent from the male profile.

For minors, the dominant keywords clustered around conditions and care-related contexts, with terms such as “myopia” and “leukemia” co-occurring with caregiving-related keywords, including “injection,” “taking medicine,” and “milk.”

The youth group displayed a heterogeneous keyword set combining lifestyle-related behaviors—such as “cola,” “eating hotpot,” and “staying up late”—with terms denoting severe acute health outcomes, including “sudden death,” “myocardial infarction,” and “esophageal cancer.”

For the middle-aged group, prominent keywords clustered around obstetric and reproductive events (eg, “premature birth,” “hemorrhage,” and “gave birth”), alongside general health maintenance terms such as “soybean” and “exercise.”

In contrast, the older adult keyword profile was dominated by pandemic-related and prevention-oriented terms, including “COVID-19,” “testing,” “vaccine,” “vaccination,” “pneumonia,” “virus,” and “infection.” Alongside these, keywords reflecting age-specific vulnerability and support contexts—such as “heatstroke,” “decline,” “function,” and “medical insurance”—were also prominent.

#### Distribution of Social Roles and Identity Labels

While gender and age provide a structural overview of demographic targeting, the distribution of social roles reveals more context-specific forms of situational vulnerability (see Multimedia Appendix 4 for the top 20 most frequent social roles). Social role mentions were distributed across several distinct categories. Generic identity labels were most prevalent, with “netizens” (n=3484) and “citizens” (n=3432) appearing most frequently.

Among specific social identities, “pregnant women” (n=1482) and “students” (n=1473) emerged as the most prominent groups, followed by “patients” (n=1298) and “parents” (n=1220).

Professional roles were also prominently represented, with “experts” (n=2659) ranking third overall and “physicians” (n=1479) appearing at high frequency. Together, these patterns suggest that, beyond generalized references to the public, health-related narratives are frequently anchored in professional authority as well as roles associated with reproduction, education, and caregiving.

### Determinants of Diffusion in Health-Related Rumor Debunking Posts

#### Validation of Sentiment Feature Extraction

To examine the potential impact of sentiment on information diffusion, we first validated the reliability of the extracted sentiment features. The internal consistency among the 3 evaluator LLMs used to construct the silver-standard dataset was substantial (ordinal α=0.694; see Multimedia Appendix 5 for comprehensive agreement metrics, including Fleiss κ and pairwise QWK). This confirms a sufficient and reliable level of consensus in evaluating the complex sentiment polarities of health-debunking discourse. Evaluated against this silver standard, the Gemma-4 annotations achieved a macro-*F*_1_-score of 0.629, an overall accuracy of 0.639, and a QWK of 0.576. Despite the inherent subjectivity and extreme semantic complexity of Weibo texts, these metrics collectively indicate a reliable and robust level of accuracy for the automated sentiment classification.

#### Predictive Performance of the XGBoost Regressors

The XGBoost regression models showed moderate explanatory power across all 3 engagement dimensions, consistent with the high inherent variability of social media engagement data. Specifically, the reposts model achieved an *R*^2^ of 0.225 (RMSE=0.344), the comments model yielded an *R*^2^ of 0.210 (RMSE=0.335), and the likes model obtained an *R*^2^ of 0.263 (RMSE=0.572). These results indicate that the models captured meaningful structural and contextual signals associated with user interactions.

#### Global Feature Importance and Divergent Engagement Associations

**Figure 5.**
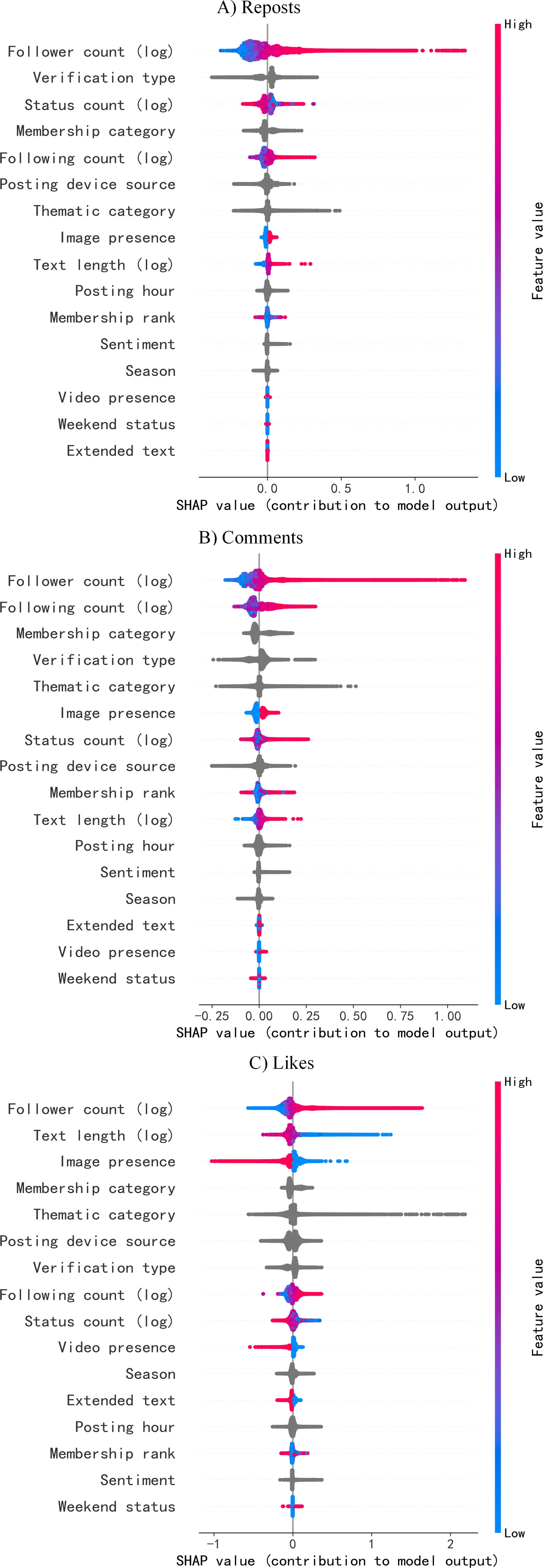
Shapley Additive Explanations (SHAP) summary plots for feature contributions to engagement predictions: (A) reposts, (B) comments, and (C) likes. Features are ranked by mean absolute SHAP value; each dot represents one post (red=high feature value, blue=low). “Extended text” is a binary indicator of whether a post exceeds 140 characters (and is thus truncated with a “show more” link); “weekend status” indicates whether the post was published on a weekend.

Figure 5 presents the SHAP summary plots for reposts, comments, and likes. Across all 3 dimensions, follower count (log-transformed) consistently ranked as the most important feature. The SHAP values indicate a direct positive association: accounts with larger follower counts were associated with higher engagement predictions, whereas accounts with smaller follower counts were associated with lower predictions.

Regarding content attributes, text length and image inclusion showed divergent associations depending on the mode of interaction. In the reposts model (Figure 5A) and comments model (Figure 5B), these features showed a positive directional association: longer texts and the presence of attached images were associated with positive marginal contributions to the predicted engagement. Conversely, this dynamic inverted in the likes model (Figure 5C): shorter texts and the absence of images were associated with higher predicted like counts. Notably, in this model, text length and image presence also emerged as the second and third most influential features, respectively, indicating that content-level attributes played a more central predictive role for likes than for the other engagement types.

Regarding other source characteristics, beyond follower count discussed earlier, the most prominent account metadata predictors differed across the 3 engagement models. In the reposts model (Figure 5A), identity verification status emerged as the second most important feature overall; its SHAP values exhibited wide dispersion on both sides of the zero axis, indicating that different verification categories were associated with either positive or negative marginal contributions to the prediction. In the same model, status count showed a predominantly negative directional association, with higher status counts corresponding to lower predicted repost magnitude. In the comments model (Figure 5B), following count emerged as the second most prominent feature overall, showing a positive association with predicted comments. In the likes model (Figure 5C), in contrast, no single additional source feature stood out as dominant, with the remaining source characteristics collectively showing moderate predictive importance.

Across all 3 models, temporal variables (season, time of day, weekend status) and text sentiment polarity consistently ranked in the lower tier of the feature hierarchy. Their SHAP values clustered tightly around the zero axis with minimal horizontal dispersion, indicating that sentiment polarity and posting time were associated with limited marginal contributions to final engagement outcomes.

#### Nonlinear Marginal Patterns of Key Predictors

**Figure 6.**
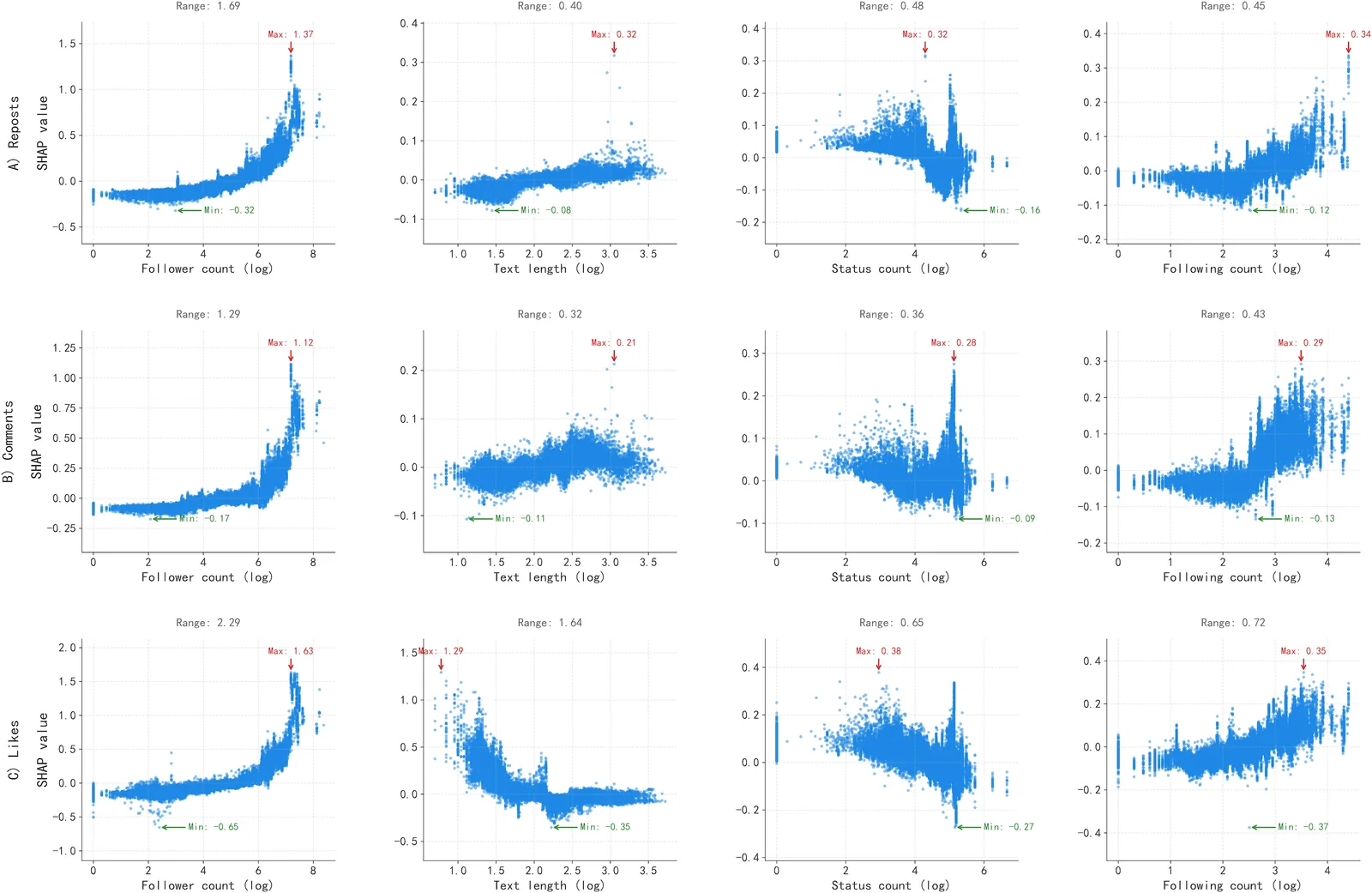
Shapley Additive Explanations (SHAP) dependence plots showing the nonlinear patterns of 4 key continuous features in XGBoost (Extreme Gradient Boosting) engagement predictions: (A) reposts, (B) comments, and (C) likes. The columns from left to right represent follower count (log-transformed), text length (log-transformed), status count (log-transformed), and following count (log-transformed). For each subplot, the x-axis represents the log-transformed feature value, and the y-axis indicates the corresponding SHAP value. Custom annotations denote the maximum (red arrows) and minimum (green arrows) SHAP values, with the total SHAP value range displayed at the top of each panel. The range is calculated using exact unrounded values, so subtraction of the displayed rounded labels may result in minor discrepancies.

Guided by their prominent roles associated with specific engagement types as identified in the summary plots, 4 continuous features—follower count, text length, status count, and following count—were selected for detailed nonlinear analysis. As illustrated in Figure 6, these dependence plots show how the marginal contribution of each feature shifts dynamically across its value distribution.

Across all 3 engagement dimensions, follower count exhibited a consistent nonlinear pattern characterized by a distinct threshold. For values below approximately 6.0, the SHAP values were predominantly distributed in the negative region with a relatively flat trajectory. Beyond this threshold, the marginal contributions demonstrated a sharp, positive upward trend. Among the 4 analyzed continuous features, follower count exhibited the widest predictive contribution range, particularly in the likes model (SHAP range≈2.3).

The dependence plots for text length revealed contrasting nonlinear patterns across the interaction types. In the likes model (Figure 6C), the highest positive SHAP values were concentrated at the lower end of the distribution, peaking at approximately 1.3 for very short texts. As text length increased, the marginal contribution exhibited a steep downward trajectory, crossing into the negative region and reaching values around −0.4. Conversely, the reposts and comments models (Figures 6A and 6B) displayed a different dynamic. After an initial dip in the shorter text range, the SHAP values transitioned into a positive upward trend. Notably, this positive trajectory in the comments model was not sustained; the main cluster of SHAP values began to decline after reaching a localized concentration in a mid-length range.

Status count demonstrated a nonlinear trajectory. At lower levels, the marginal contributions were predominantly positive. As the post count increased, these values declined into the negative region to form a distinct trough, before exhibiting a rebound trend at the higher end of the distribution. Notably, the onset of this downward shift occurred earlier in the likes model compared to reposts and comments.

Finally, following count exhibited a clear threshold pattern. At the lower end of the distribution, the SHAP values were predominantly negative. However, as the values surpassed a mid-range threshold (approximately 2.0-2.5 on the logarithmic scale), the scatter plots crossed the zero axis and transitioned into a positive trajectory for larger following counts.

## Discussion

### Principal Results

#### Overview

By analyzing a comprehensive dataset spanning 15 years of health-related rumor debunking posts on Sina Weibo, this study characterizes the longitudinal evolution, thematic diversity, demographic representations, and engagement predictors of corrective communication in China’s digital health ecosystem. Our findings demonstrate that health-related rumor debunking on Weibo was strongly influenced by major public health crises, particularly during the COVID-19 pandemic. At the same time, the topical landscape has progressively diversified over the past decade, with lifestyle-related and well-being–related themes showing relatively stable long-term growth. In addition, although most debunking posts targeted the general public, clear differences emerged across gender and age groups in both representation and associated health concerns, and these demographic references were unevenly linked to specific health topics. Finally, interpretable machine learning indicated that engagement was associated primarily with source-level reach, with follower count being the strongest predictor across reposts, comments, and likes, while the contribution of content attributes varied by interaction type and was most pronounced for likes; sentiment and posting time showed limited associations. Considered jointly, these findings reveal a structural asymmetry in the debunking ecosystem that single-dimension analyses would not capture: the content production side is maturing, as thematic coverage is broadening and demographic differentiation is emerging, while the distribution side remains governed by source-level reach largely irrespective of content attributes. This content distribution misalignment means that advances in what is corrected and for whom do not automatically translate into proportional communicative impact.

#### Crisis-Driven Evolution and Subsequent Attention Rebalancing

Our longitudinal findings (2009‐2024) suggest that the COVID-19 pandemic was associated with substantial shifts in the volume of health rumor debunking on Sina Weibo, with effects that appear to have extended well beyond the acute outbreak period. The sharp escalation of health-related debunking activity in 2020 likely reflected the convergence of 2 parallel dynamics. On the one hand, the outbreak generated unprecedented levels of misinformation under conditions of high public uncertainty, concerning transmission routes, protective measures, vaccines, and treatment strategies. On the other hand, the pandemic simultaneously triggered an intensified institutional response, as government agencies and platforms rapidly expanded corrective communication efforts to stabilize the online information environment during a large-scale public health emergency.

Beyond this initial surge, the elevated level of health-related debunking activity persisted well past the most acute outbreak stages. Even after the initial outbreak phase (2020) subsided, the volume of health-related debunking remained substantially elevated throughout 2020 to 2023 compared with the prepandemic baseline. This pattern suggests that the pandemic may have functioned not only as a short-term focusing event but also as a catalyst for broader governance reconfiguration. Institutional mechanisms initially mobilized under emergency conditions—including specialized debunking accounts, cross-platform coordination, and intensified administrative intervention—appear to have become partially embedded within routine governance practices. This pattern can be interpreted through the broader lens of Punctuated Equilibrium Theory [62], which proposes that exogenous shocks can disrupt previously stable policy equilibria and generate durable shifts in governance arrangements. However, this analysis tracks volumes of debunking posts rather than the institutional arrangements themselves; therefore, this interpretation should be regarded as suggestive rather than conclusive.

At the same time, this sustained postpandemic elevation should not be interpreted solely as evidence of continuously heightened public concern regarding health misinformation. The persistence of debunking activity may also reflect the temporary continuation of institutional mandates and governance infrastructures established during the emergency phase. In other words, the prolonged visibility of health rumor governance likely emerged through the interaction between enduring public sensitivity and sustained administrative mobilization.

By 2024, however, the relative share of health-related debunking declined markedly, despite the overall volume of rumor refutation posts reaching its historical peak. Importantly, this decline does not necessarily indicate a weakening of misinformation governance capacity. Rather, it may reflect a redistribution of institutional attention and potentially of broader public salience as pandemic-related risks gradually lost their exceptional status and competing social issues regained visibility within the broader governance agenda. Alternative explanations—including shifts in official governance mandates and potential changes in platform-level recommendation algorithms that may influence content visibility—also warrant consideration, although the present dataset does not allow us to formally separate these contributing factors. This mechanism is broadly consistent with the issue attention cycle [63], which suggests that highly salient public problems gradually lose centrality as crisis urgency fades and attention shifts toward emerging concerns. As with Punctuated Equilibrium Theory mentioned earlier, this framework is offered as an interpretive lens rather than a directly tested mechanism, given that our data capture debunking activity rather than the underlying processes of public attention themselves.

Together, these findings outline a dynamic cycle of crisis-driven rumor governance characterized by emergency escalation, partial institutionalization, and subsequent rebalancing of attention across issue domains.

#### State-Centered Governance in Health Rumor Debunking

Our findings reveal a predominantly state-centered configuration in China’s health rumor debunking ecosystem. Government-verified accounts contributed nearly two-thirds (64.69%) of all health-related debunking posts, substantially exceeding the combined output of media, institutional, and enterprise accounts. While the share of government accounts was statistically higher in health-related debunking than in non–health-related debunking (chi-square test, *P*<.001), the effect size was small (Cramér *V*=0.05), indicating that government dominance is a general feature of Sina Weibo’s debunking ecosystem rather than a phenomenon unique to the health domain. Nonetheless, this dominance is amplified within the health context: the government-to-media output ratio reached approximately 4.3:1 in health-related debunking, compared with 2.5:1 in non–health-related debunking. This contrast suggests that, although state actors play a leading role across topic domains, their relative weight is even more pronounced when the corrective communication concerns health.

This configuration is consistent with Media System Dependency Theory [64], which posits that audience reliance on particular information sources intensifies under conditions of uncertainty and perceived risk. As health rumors often involve scientific complexity and potential collective consequences—such as panic buying, vaccine hesitancy, or heightened public anxiety—commercially oriented media, shaped by market incentives and attention dynamics, may be less inclined to function as definitive arbiters of truth. State actors, in contrast, are institutionally positioned to supply administrative authority and offer authoritative reference points that the public can draw upon when navigating conflicting health claims online.

This state-centered pattern, however, should not be read as a universal template for misinformation governance. Comparative evidence from Canada and the United States points to a more distributed, multiactor model, in which corrective communication is shared across public health agencies (eg, the Centers for Disease Control and Prevention and Health Canada), independent fact-checking organizations, professional medical associations, and academic experts, rather than concentrated within central government accounts [65,66]. Two factors may help explain this divergence. First, in contexts where institutional trust is fragmented along political or ideological lines, corrective messages delivered through a diverse set of nongovernmental and quasi-governmental actors may achieve broader credibility than messages issued directly by central authorities [67,68]. Second, in such settings, direct government involvement in online information governance can itself become politically contested, which makes distributed governance both a normative preference and a pragmatic strategy [69]. Overall, these contrasts indicate that effective debunking architectures are shaped by broader institutional and sociopolitical contexts and that the Chinese pattern documented here reflects one viable configuration rather than a generalizable blueprint.

#### Thematic Evolution: From Acute Survival Concerns to Everyday Well-Being

The thematic evolution of health rumor debunking suggests a gradual broadening of public attention from acute crisis-related concerns toward a wider range of everyday health and well-being issues. This shift plausibly reflects the increasing ubiquity of social media and broader changes in how health information is accessed, circulated, and interpreted online [70]. Within this maturing digital environment, we observe a clear divergence in temporal trajectories across topic types. Event-driven topics, such as vaccines and personal protective equipment, tend to follow an “explosive but transient” pattern, with sharp spikes closely aligned with major epidemic phases. In contrast, lifestyle-oriented topics, most notably vision and eye health, as well as nutrition, exhibit more sustained growth over time, suggesting enduring relevance beyond discrete crisis periods.

This observed reorientation of public attention may be interpreted through the lens of Maslow’s hierarchy of needs [71]. During the height of the pandemic, public attention was disproportionately concentrated on safety-related concerns, including infection avoidance and immediate risk reduction. As the perceived severity of the threat declined, attention appears to have gradually expanded toward issues associated with everyday functioning and quality-of-life management.

At the same time, the sustained prominence of lifestyle-related rumors can be understood in relation to the “medicalization of daily life” [72], whereby ordinary behaviors—such as screen use, diet, and sleep—are increasingly framed in medicalized terms that invite expert-like intervention. Within this context, routine health anxieties may accumulate over time, generating ongoing demand for ostensibly “scientific” optimization and creating a fertile environment for pseudoscientific claims, dietary myths, and “quick-fix” narratives. The coexistence of these distinct temporal archetypes suggests that effective debunking calls for simultaneously managing acute crisis response and sustained routine correction, a duality whose practical implications are further shaped by how corrections circulate and reach their audiences.

#### Demographic Differentiation in Health Narratives: Patterns Across Gender, Age, and Social Roles

Our demographic analysis suggests that digital health narratives are not neutral but systematically differentiated along gender and age lines. To interpret these disparities, we draw on several sociopsychological frameworks while remaining attentive to the boundaries of our data: because our dataset captures keyword-level textual representations rather than direct psychometric or behavioral measures, we frame these theoretical insights as exploratory interpretations rather than definitive causal mechanisms. In parallel, we situate these narrative differences within the structural realities of the digital environment—platform demographics, algorithmic biases, and contemporary digital culture.

With respect to gender, female-related mentions (n=5352, 5.71%) substantially outnumbered those associated with men (n=2799, 2.98%), and the female-associated keyword profile was dominated by terms related to reproductive and maternal health (eg, “uterus,” “ovary,” and “pregnancy”). This patterned framing could tentatively be interpreted through the lens of Objectification Theory [73], which highlights how women’s bodies are often rendered salient through appearance- and function-oriented discourses. A more direct alternative explanation lies in the engagement-driven algorithmic structure of social media. Prior research consistently demonstrates that women are more active seekers and sharers of online health information, particularly regarding maternal and routine well-being [74-76]. Drawing on this prior evidence, one possibility is that platform algorithms—which prioritize content with high interaction rates—disproportionately amplify reproductive and appearance-oriented debunking content because these topics are documented to generate engagement from female demographics. We note that this remains an inference drawn from external literature rather than from engagement metrics within our own dataset.

In contrast, male-related keywords clustered around 2 distinct categories: acute and high-severity health events (eg, “cardiac arrest” and “choking”) and pandemic control terms (eg, “lockdown” and “quarantine”). While the acute-event cluster resonates with tentative insights from Hegemonic Masculinity theory [77]—which has been used to explain the relative invisibility of routine male health concerns—it may also reflect the interaction between gendered health information behaviors and platform visibility dynamics. Prior research suggests that men generally engage less with routine or preventive health information online [78]. Drawing on prior platform studies, low-interaction topics may be less likely to receive algorithmic amplification [79], whereas acute and emotionally arousing medical emergencies are more commonly associated with rapid sharing and public attention [5]. Within this framework, routine male health concerns may remain relatively less visible while acute medical events become more prominent in rumor debunking discussions. The co-occurrence of pandemic control terms in the male-associated profile is plausibly a separate phenomenon, reflecting the broader prominence of these topics during the COVID-19 period rather than a gender-specific pattern. As mentioned earlier, these interpretations rest on external evidence rather than on engagement data within our own corpus.

Across the life course, vulnerability profiles further diverge. Youth emerged as the most frequently referenced group (n=5020, 5.35%), a pattern that closely mirrors Weibo’s user demographics, where young adults constitute the platform’s core population [80]. Beyond frequency, the youth-associated keyword profile was notably heterogeneous, including both lifestyle-related behaviors—such as “cola,” “hotpot,” and “staying up late”—and terms denoting severe acute health outcomes, such as “sudden death,” “myocardial infarction,” and “esophageal cancer.” This heterogeneous profile likely reflects the breadth of health concerns relevant to Weibo’s predominantly young user base, encompassing both everyday lifestyle topics and the acute medical incidents that periodically attract public attention in this demographic.

Minors also appeared as a highly visible group (n=4878, 5.20%), reflecting their socially recognized vulnerability and the prioritization of child protection in public discourse. The prominence of caregiving-related keywords (eg, “injection,” “taking medicine,” and “milk”) alongside the high frequency of the “parents” social role (n=1220) suggests that minors’ health concerns in this corpus are frequently mediated through caregiver discourse rather than appearing as self-expressed concerns. In line with prior research [81], this pattern may reflect parental proxy health information seeking, in which caregivers actively engage with health-related content on behalf of children—although our keyword-level data describe textual co-occurrence rather than directly capturing information-seeking behaviors. Correspondingly, the discourse is heavily concentrated on “vision and eye health” (Figure 4) and keywords such as “myopia” and “leukemia” (Multimedia Appendix 3), reflecting both routine developmental concerns and heightened anxieties surrounding severe pediatric conditions. This pattern is further reinforced by the prominence of the “student” social role (n=1473), suggesting that health narratives about minors are frequently framed within educational settings rather than purely biological trajectories of development.

Moving further along the life course, the middle-aged group exhibits a distinctive and multifaceted keyword profile, combining high-risk obstetric terms (eg, “premature birth,” “hemorrhage,” and “gave birth”) with general health maintenance terms (eg, “soybean” and “exercise”). The presence of obstetric terms potentially indicates that reproductive risk remains a salient concern in middle-aged discourse, a pattern that may be situated within broader demographic shifts in contemporary China, including the growing prevalence of pregnancies at advanced maternal age following the relaxation of birth control policies under the 2- and 3-child initiatives. We note, however, that the present cross-sectional data do not directly link this keyword pattern to those policy shifts. The co-occurrence of health maintenance terms further suggests that middle-aged health discourse is not exclusively framed around acute reproductive risks but also encompasses general well-being concerns.

By contrast, the older adult group (n=3590) exhibited the lowest overall frequency among the defined age groups, although the gap relative to the other groups was modest. Rather than reflecting outright digital marginalization, this lower frequency may partly arise because older adults are often represented by others rather than through direct self-expression—a pattern that, although different in form, parallels the proxy-mediated representation observed for minors above, and is consistent with prior research on age-related disparities in social media use [75]. The older adult–associated keyword profile was dominated by pandemic-related terms (eg, “COVID-19,” “vaccine,” “pneumonia,” and “infection”), reflecting the prominence of older adults as a high-risk population during the COVID-19 period. Alongside these, keywords such as “heatstroke,” “decline,” “function,” and “medical insurance” suggest that older adult health narratives in this corpus also tend to be framed around environmental risks and institutional support, although our data cannot directly speak to the underlying agency dynamics.

In parallel with these demographic patterns, the high frequency of “experts” (n=2659) and “physicians” (n=1479) in the overall corpus points to the co-occurrence of professional roles alongside demographic identity labels within rumor debunking discourse. Although our keyword-level data do not directly distinguish the discursive functions these mentions serve, this co-occurrence pattern is broadly consistent with prior observations of expert-driven health communication, in which professional voices and lay subjects of health concern jointly populate corrective discourse.

Although the majority of health rumor debunking posts in our corpus address a general audience without explicitly mentioning specific groups, the distinct patterns observed within the targeted subset suggest the potential value of precision communication. For health communication practitioners, these differentiated findings indicate that governance efforts might be optimized by supplementing standard universal broadcasts with tailored messages. When addressing niche health concerns, configuring the framing to the distinct contexts of specific genders, age groups, and social roles could enhance the relevance of corrective information. Across these patterns, we emphasize that our analysis describes systematic textual co-occurrences within rumor debunking discourse and that the underlying mechanisms—whether psychological, cultural, or algorithmic—warrant direct empirical testing in future research.

#### Predictors of Engagement: Source Hierarchy and Differentiated Engagement Modes

Our engagement analysis suggests a partial “source-over-content” tendency, particularly for diffusion (reposts) and interactive engagement (comments): the spread of debunking messages appears to be more strongly associated with who delivers the message than with what the message is about. Across all 3 engagement types, follower count (operationalized as the log-transformed follower count) consistently emerged as the dominant predictor, while sentiment and posting time showed comparatively limited predictive contributions. One plausible interpretation is that, in information-saturated social media environments, users often lack the time, cognitive resources, or domain-specific expertise required to systematically evaluate complex health information. Under such conditions, they may rely on readily available peripheral cues—such as audience size and verification-related signals—as heuristic indicators of credibility and relevance. This behavioral pattern is consistent with the Elaboration Likelihood Model, which describes the role of peripheral processing when motivation or ability for deep evaluation is limited [82]. We note, however, that our data permit only predictive associations and cannot directly test the underlying cognitive mechanisms; the Elaboration Likelihood Model is therefore offered as one plausible interpretive lens rather than a tested explanation.

Crucially, the associations of source and content features diverged depending on the mode of engagement. For reposts, follower count was followed by institutional verification (verified type) as the next most prominent source predictor, suggesting that diffusion breadth is more strongly associated with high-status, institutionally credentialed sources than with the substantive content of the post. For comments, in contrast, following count emerged as the second most prominent source predictor and was positively associated with predicted comments. One possible interpretation is that accounts following many others tend to be more active social participants, whose posts may correspond to environments more conducive to dialogue. Social exchange theory [83] offers a complementary interpretive frame in which reciprocal networking signals may correspond to lower psychological thresholds for audience response [84], but this interpretation should be treated as exploratory given the observational nature of our data.

The most striking departure from this source-dominant pattern appeared in the likes model. Although follower count remained the leading predictor, text length and image inclusion rose to become the second and third most prominent features, with shorter texts and the absence of images associated with higher predicted likes. This pattern suggests that the lightweight, low-effort nature of liking may render lightweight content (brief, text-only posts) more compatible with the cognitive and behavioral cost of the engagement act itself. For likes, therefore, content-level attributes appear to play a more central predictive role than for reposts or comments, partially qualifying the broader “source-over-content” framing.

Despite these mode-specific nuances, the overarching ecosystem remains marked by a pronounced concentration pattern reminiscent of the Matthew Effect [85]. The consistent dominance of follower count across all 3 engagement metrics, together with the predominantly negative SHAP values observed for low-follower accounts, suggests a cold-start dynamic in which accounts lacking sufficient initial audience size struggle to gain visibility. Importantly, this pattern need not reflect user-level cognitive heuristics alone; it may also be shaped substantially by platform-level algorithmic mechanisms. On Weibo, recommendation and ranking algorithms tend to amplify content from accounts that have already accumulated engagement, producing a feedback loop in which visibility begets further visibility. The “source-over-content” pattern we observe in the data is therefore likely a joint product of user heuristics and platform algorithmic design, rather than a pure reflection of either mechanism alone. Without access to platform algorithmic data, we cannot fully disentangle these 2 contributing pathways, and we offer this interpretation as a hypothesis warranting further investigation.

Together, these patterns suggest a visibility disadvantage for credible but less-followed sources. As the SHAP dependence plots show, accounts with follower counts below approximately 10⁶ were associated with predominantly negative marginal contributions across all 3 engagement dimensions, with a sharp positive inflection emerging only beyond this threshold. Credible but low-resource voices may therefore remain structurally below diffusion thresholds regardless of content quality, implying that effective governance requires not only producing accurate corrections but also enabling their circulation. Platform-level interventions, such as algorithmic amplification mechanisms or endorsement features that boost the visibility of credible grassroots sources, may help ensure that evidence-based correction is not structurally suppressed by deficits in social capital.

### Limitations

First, contextual generalizability is constrained by the sociopolitical setting of this study. Our analysis is based on Sina Weibo within China’s specific information governance framework. While this context offers a valuable lens into a relatively state-centered debunking ecology, it may limit the transferability of our findings to other institutional environments. In particular, the administrative-led configuration observed here differs from the more distributed, multiactor models discussed earlier, limiting the extent to which our findings generalize to such settings. Future comparative research across political and regulatory systems is needed to examine how governance structures shape the organization and effectiveness of rumor debunking.

Second, platform specificity may restrict the applicability of our conclusions beyond Weibo’s media environment. Sina Weibo functions largely as a text-based public sphere with repost-centered diffusion dynamics. In contrast, emerging platforms such as Douyin (the Chinese counterpart of TikTok) operate with different content formats and recommendation architectures, while WeChat (Tencent) represents a more closed, private-domain communication system. Future work should adopt cross-platform designs to evaluate how media format, network structure, and algorithmic logic jointly influence the visibility and diffusion of debunking content.

Third, the corpus may be incomplete because of our reliance on keyword-based retrieval at the construction stage. Specifically, using “rumor debunking” (辟谣) as the primary query term may omit corrective messages that perform fact-checking implicitly without explicit debunking labels (eg, physicians providing corrective explanations in routine health guidance). Future research could incorporate semantic dense retrieval methods or iterative active learning pipelines to capture a broader spectrum of implicit corrective communication and improve coverage of grassroots correction practices.

Fourth, our engagement data are observational, and the SHAP attributions therefore characterize predictive contributions rather than causal effects. Although our models incorporated a comprehensive set of source, content, and contextual features, unobserved factors—such as platform-level recommendation weights or offline amplification events—may still shape diffusion dynamics. Future studies should use experimental designs or formal causal inference techniques to directly isolate the mechanisms underlying user engagement.

### Conclusions

By analyzing 15 years of health-related rumor debunking posts on Sina Weibo (2009‐2024), this study offers a longitudinal account of official corrective communication along 3 dimensions: its thematic evolution, the populations it references, and the factors associated with its diffusion. First, the focus of health-related debunking broadened over time, moving from acute crises—most notably COVID-19—toward a wider range of routine health and well-being concerns. Second, most debunking posts were not directed at any specific demographic group; among the minority that explicitly referenced particular populations, references were unevenly distributed across gender and age and were linked to distinct health concerns. Third, our predictive analysis indicates that engagement was associated primarily with source-level reach: follower count was the strongest predictor of reposts, comments, and likes alike, outweighing the substance of the correction. Beyond this shared pattern, the secondary predictors differed across the 3 interaction types, indicating that reposts, comments, and likes are shaped by partly distinct configurations of source and content attributes rather than by a single uniform mechanism.

Viewed jointly, these 3 dimensions reveal a structural asymmetry in the debunking ecosystem: the content side is maturing in both thematic scope and demographic differentiation, while distribution remains governed by source-level reach largely irrespective of content attributes. This asymmetry carries specific governance implications. First, the coexistence of event-driven spikes and sustained growth trajectories among health-related debunking topics indicates that crisis response capacity alone is insufficient; building long-term, routine communication capacity for lifestyle-related concerns that are steadily gaining prominence may be equally important. Second, the systematic associations between demographic groups and specific health concerns, such as reproductive health terms in female-associated content and pandemic-related terms in older adult-associated content, provide an empirical baseline that practitioners can draw upon to assess the coverage and priorities of current debunking efforts. Third, the SHAP analysis revealed a clear threshold effect for follower count, below which accounts were associated with predominantly negative marginal contributions to engagement, suggesting that credible but low-resource voices face a structural diffusion barrier; platform-level mechanisms that help such sources circulate corrections may therefore be an essential complement to relying on established authoritative accounts alone.

## Supplementary material

10.2196/91516 Multimedia Appendix 1 Validation metrics for the information extraction framework (interannotator agreement, overall model performance, and category-specific performance evaluation for gender and age variables).

10.2196/91516 Multimedia Appendix 2 Detailed statistics of gender and age group mentions across the top 10 BERTopic-derived themes.

10.2196/91516 Multimedia Appendix 3 Top 20 characteristic keywords of health-related rumor debunking posts, stratified by gender and age.

10.2196/91516 Multimedia Appendix 4 Top 20 most frequent social roles identified in health-related rumor debunking posts.

10.2196/91516 Multimedia Appendix 5 Intermodel agreement and performance metrics for sentiment classification.

## Abbreviations

BERT: Bidirectional Encoder Representations from Transformers
HDBSCAN: Hierarchical Density-Based Spatial Clustering of Applications with Noise
IAA: interannotator agreement
LLM: large language model
PPE: personal protective equipment
QWK: Quadratically Weighted Cohen κ
RMSE: root-mean-squared error
RQ: research question
SetFit: Sentence Transformer Fine-tuning
SHAP: Shapley Additive Explanations
XGBoost: Extreme Gradient Boosting
