# Supplementary material for: Health-Related Rumor Debunking on Sina Weibo in China (2009-2024): 15-Year Retrospective Infodemiology Study
Source: J Med Internet Res. 2026 Sep 10;28:e91516. doi: 10.2196/91516 (PMC13561439; doi:10.2196/91516)
Supplement: Supplementary file 1 [file jmir-v28-e91516-s001.docx]

**Validation metrics for the information extraction framework (Inter-annotator agreement, overall model performance, and category-specific performance evaluation for gender and age variables)**

Table S1. Inter-annotator agreement metrics for the construction of the gold-standard dataset.

| Target Variable | Metric | Score |
| --- | --- | --- |
| Gender (Extraction) | Avg Set-F1 | 0.967 |
| Gender (Normalization) | Macro-Avg Alpha | 0.820 |
| Age (Extraction) | Avg Set-F1 | 0.949 |
| Age (Normalization) | Macro-Avg Alpha | 0.824 |
| Social Roles | Avg Set-F1 | 0.831 |
| Health Content | BERTScore F1 | 0.876 |

Table S2. Overall performance evaluation of the Large Language Model (DeepSeek-V3.2) against the human-annotated gold standard.

| Target Variable | Metric | Score |
| --- | --- | --- |
| Gender (Extraction) | Set-F1 | 0.955 |
| Gender (Normalization) | Macro-F1 | 0.802 |
| Age (Extraction) | Set-F1 | 0.926 |
| Age (Normalization) | Macro-F1 | 0.701 |
| Social Roles | Set-F1 | 0.747 |
| Health Content | BERTScore | 0.749 |

Table S3. Category-specific performance evaluation of the Large Language Model (DeepSeek-V3.2) for gender and age extraction against the human-annotated gold standard.

| Demographic Dimension | Category | Precision | Recall | F1-Score | Support (n) |
| --- | --- | --- | --- | --- | --- |
| Gender | Female | 0.842 | 0.800 | 0.821 | 20 |
|  | Male | 0.692 | 0.900 | 0.783 | 10 |
| Age Group | Minor (<18 years) | 0.833 | 0.938 | 0.882 | 16 |
|  | Youth (18–44 years) | 0.692 | 0.750 | 0.720 | 12 |
|  | Middle-aged (45–59 years) | 0.571 | 0.500 | 0.533 | 8 |
|  | Older adults (≥60 years) | 0.500 | 1.000 | 0.667 | 3 |

Note. The 200 gold-standard posts were randomly sampled from the corpus. Because the majority of debunking posts address a general audience without referencing specific demographic groups, the Support values reflect the natural distribution of demographic mentions rather than a balanced sampling design. Metrics for categories with small Support (e.g., Older adults, n = 3) should be interpreted with caution.
