# Supplementary material for: Health-Related Rumor Debunking on Sina Weibo in China (2009-2024): 15-Year Retrospective Infodemiology Study
Source: J Med Internet Res. 2026 Sep 10;28:e91516. doi: 10.2196/91516 (PMC13561439; doi:10.2196/91516)
Supplement: Supplementary file 2 [file jmir-v28-e91516-s002.docx]

**Detailed statistics of gender and age group mentions across the top 10 BERTopic-derived themes**

Table S1. Frequency distribution of gender mentions by topic.

| **Topic** | **Male** | **Female** | **Excluded** |
| --- | --- | --- | --- |
| 0 | 7 | 17 | 1 |
| 1 | 292 | 195 | 67 |
| 2 | 5 | 49 | 4 |
| 3 | 1 | 4 | 0 |
| 4 | 0 | 1 | 3 |
| 5 | 4 | 0 | 1 |
| 6 | 4 | 11 | 3 |
| 7 | 5 | 0 | 2 |
| 8 | 2 | 21 | 2 |
| 9 | 1 | 14 | 14 |
| Total | 321 | 312 | 97 |

Note. Values represent the number of posts mentioning specific demographic keywords within each topic. To ensure distinct categorization, the “Excluded” column in Table S1 accounts for posts that mentioned both male and female terms simultaneously.

Table S2. Frequency distribution of age group mentions by topic.

| **Topic** | **Minor** | **Youth** | **Middle-aged** | **Older adults** | **Excluded** |
| --- | --- | --- | --- | --- | --- |
| 0 | 29 | 13 | 7 | 12 | 2 |
| 1 | 20 | 116 | 44 | 36 | 0 |
| 2 | 17 | 33 | 9 | 7 | 1 |
| 3 | 13 | 38 | 29 | 31 | 2 |
| 4 | 3 | 1 | 9 | 9 | 1 |
| 5 | 5 | 5 | 4 | 1 | 0 |
| 6 | 7 | 12 | 3 | 0 | 0 |
| 7 | 13 | 4 | 2 | 4 | 1 |
| 8 | 10 | 19 | 10 | 10 | 0 |
| 9 | 102 | 126 | 113 | 170 | 3 |
| Total | 219 | 367 | 230 | 280 | 10 |

Note. Values represent the number of posts mentioning specific demographic keywords within each topic. To ensure distinct categorization, the “Excluded” column in Table S2 accounts for posts that mentioned all defined age groups simultaneously. The specific gender or age columns count posts mentioning only that respective category.
