# Supplementary material for: Health-Related Rumor Debunking on Sina Weibo in China (2009-2024): 15-Year Retrospective Infodemiology Study
Source: J Med Internet Res. 2026 Sep 10;28:e91516. doi: 10.2196/91516 (PMC13561439; doi:10.2196/91516)
Supplement: Supplementary file 3 [file jmir-v28-e91516-s003.docx]

**Top 20 characteristic keywords of health-related rumor-debunking posts, stratified by gender and age**

Table S1. Frequency ranking of characteristic keywords by gender and age.

| Demographic Category | Subgroup | Top Keywords (Ranked by Prominence) |
| --- | --- | --- |
| **Gender** | **Male** | Pandemic, COVID-19, pneumonia, testing, infection, nucleic acid, positive, novel coronavirus, prevention and control, virus, health, lockdown, heart, rumors, cardiac arrest, confirmed cases, choking, quarantine, cases, epidemic-related |
|  | **Female** | Pregnant women, pandemic, COVID-19, infection, pneumonia, uterus, ovary, vaccination, health, pregnancy, gestational period, testing, nutrition, nucleic acid, fetus, pregnancy brain, three years, female, vaccine, positive |
| **Age Group** | **Minor** | Children, myopia, child, COVID-19, vaccine, infection, pneumonia, illness, pandemic, leukemia, vaccination, taking medicine, injection, quick recovery, testing, health, treatment, common cold, milk, nucleic acid |
|  | **Youth** | COVID-19, pandemic, pneumonia, confirmed cases, health, infection, external auditory canal, positive, prevention and control, sudden death, esophageal cancer, staying up late, cases, testing, quarantine, long-term, myocardial infarction, cola, eating hotpot, virus |
|  | **Middle-aged** | Pregnant women, hemorrhage, premature birth, successfully, pandemic, gave birth, a pair, son and daughter, soybean, sudden death, infection, exercise, pneumonia, nucleic acid, coronavirus, testing, protein, fiber, sent to hospital, isoflavones |
|  | **Older adults** | COVID-19, health, pandemic, testing, vaccine, disease, vaccination, pneumonia, nucleic acid, virus, infection, older adults, re-positive, prevention, seniors, heatstroke, medical insurance, function, prevention and control, decline |

Note. Keywords are listed in descending order of frequency based on the text mining results. Terms were translated from the original Chinese text.
