# Supplementary material for: Health-Related Rumor Debunking on Sina Weibo in China (2009-2024): 15-Year Retrospective Infodemiology Study
Source: J Med Internet Res. 2026 Sep 10;28:e91516. doi: 10.2196/91516 (PMC13561439; doi:10.2196/91516)
Supplement: Supplementary file 4 [file jmir-v28-e91516-s004.docx]

**Top 20 most frequent social roles identified in health-related rumor debunking posts**

Table S1. Frequency ranking of social roles.

| **Rank** | **Chinese Term (Original)** | **English Translation** | **Frequency** |
| --- | --- | --- | --- |
| 1 | 网民 | Netizens | 3,484 |
| 2 | 市民 | Citizens | 3,432 |
| 3 | 专家 | Experts | 2,659 |
| 4 | 网友 | Internet Users | 1,880 |
| 5 | 孕妇 | Pregnant Women | 1,482 |
| 6 | 医生 | Physicians | 1,479 |
| 7 | 学生 | Students | 1,473 |
| 8 | 患者 | Patients | 1,298 |
| 9 | 家长 | Parents | 1,220 |
| 10 | 造谣者 | Rumormongers | 864 |
| 11 | 消费者 | Consumers | 782 |
| 12 | 居民 | Residents | 746 |
| 13 | 职工 | Workers/Staff | 602 |
| 14 | 记者 | Journalists | 599 |
| 15 | 员工 | Employees | 534 |
| 16 | 核酸检测阳性患者 | Nucleic Acid Test Positive Patients | 529 |
| 17 | 警方 | Police | 381 |
| 18 | 糖尿病患者 | Diabetic Patients | 375 |
| 19 | 教授 | Professors | 367 |
| 20 | 确诊病例 | Confirmed Cases | 340 |
