# Supplementary material for: Health-Related Rumor Debunking on Sina Weibo in China (2009-2024): 15-Year Retrospective Infodemiology Study
Source: J Med Internet Res. 2026 Sep 10;28:e91516. doi: 10.2196/91516 (PMC13561439; doi:10.2196/91516)
Supplement: Supplementary file 5 [file jmir-v28-e91516-s005.docx]

**Inter-Model Agreement and Performance Metrics for Sentiment Classification**

Table S1. Inter-model agreement metrics for silver-standard dataset construction.

| **Evaluation Metric** | **Agreement Score** |
| --- | --- |
| **Overall Agreement** |  |
| Ordinal Krippendorff's Alpha | 0.6938 |
| Fleiss' Kappa | 0.6541 |
| **Pairwise Agreement (QWK)** |  |
| Doubao vs. Kimi | 0.6326 |
| Doubao vs. Qwen | 0.6966 |
| Kimi vs. Qwen | 0.6362 |

Table S2. Overall performance of Gemma4:e4b on sentiment classification against the silver-standard (*N*=1,500)

| **Evaluation Metric** | **Score** |
| --- | --- |
| Quadratically Weighted Cohen's Kappa (QWK) | 0.5763 |
| Accuracy | 0.6387 |
| Macro-F1 | 0.6290 |
| Weighted-F1 | 0.6484 |
